# Comparative Genome Analysis of Three *Komagataeibacter* Strains Used for Practical Production of Nata-de-Coco

**DOI:** 10.3389/fmicb.2021.798010

**Published:** 2022-02-04

**Authors:** Koji Ishiya, Hideki Kosaka, Takashi Inaoka, Keitarou Kimura, Nobutaka Nakashima

**Affiliations:** ^1^Bioproduction Research Institute, National Institute of Advanced Industrial Sciences and Technology (AIST), Sapporo, Japan; ^2^Research and Development Department, Fujicco Co., Ltd., Chuo-ku, Japan; ^3^Institute of Food Research, National Agriculture and Food Research Organization (NFRI/NARO), Tsukuba, Japan

**Keywords:** cellulose, comparative genome analysis, duplicated sequences, *Komagataeibacter*, nata-de-coco production

## Abstract

We determined the whole genome sequences of three bacterial strains, designated as FNDCR1, FNDCF1, and FNDCR2, isolated from a practical nata-de-coco producing bacterial culture. Only FNDCR1 and FNDCR2 strains had the ability to produce cellulose. The 16S rDNA sequence and phylogenetic analysis revealed that all strains belonged to the *Komagataeibacter* genus but belonged to a different clade within the genus. Comparative genomic analysis revealed cross-strain distribution of duplicated sequences in *Komagataeibacter* genomes. It is particularly interesting that FNDCR1 has many duplicated sequences within the genome independently of the phylogenetic clade, suggesting that these duplications might have been obtained specifically for this strain. Analysis of the cellulose biosynthesis operon of the three determined strain genomes indicated that several cellulose synthesis-related genes, which are present in FNDCR1 and FNDCR2, were lost in the FNDCF1 strain. These findings reveal important genetic insights into practical nata de coco-producing bacteria that can be used in food development. Furthermore, our results also shed light on the variation in their cellulose-producing abilities and illustrate why genetic traits are unstable for *Komagataeibacter* and *Komagataeibacter*-related acetic acid bacteria.

## Introduction

Numerous bacteria produce cellulose extracellularly. Bacterial cells are often entrapped in the cellulose fiber, forming pellicle at the air–liquid interface. Depending on the bacterial strain and culture conditions, cells consume as much as 10% of the total energy budget for cellulose biosynthesis (Ross et al., 1991; Chawla et al., 2009). Although the reason for forming pellicle remains controversial, bacterial cells in the pellicle might be able to yield oxygen more efficiently than planktonic cells in liquid (Lee et al., 2014). Bacterial cellulose is expected to offer great potential for use in biomedical and food industries because of its high water-holding capacity, ultrafine network structure, biocompatibility, and high tensile strength in a wet state (Londrina et al., 2018).

A well-known example of bacterial cellulose in industry is nata-de-coco, an indigenous jelly-like food of Southeast Asia. The fibrous base of nata-de-coco, which consists of pure bacterial cellulose, is produced industrially through fermentation of coconut water using acetic acid bacteria (AAB). Among AAB, the species of *Komagataeibacter* genus (also known as *Gluconacetobacter* genus), which are kinds of α-proteobacteria and obligately aerobic and acidophilic bacteria, are the most preferably employed practically because the bacteria of this genus have the exceptionally high capability for producing cellulose (Ryngajłło et al., 2019).

However, the productivity of nata-de-coco by these bacteria is highly unstable; AAB are well known to be genetically unstable and to lose cellulose producing ability frequently in the absence of selection pressures (Matsutani et al., 2015; Ryngajłło et al., 2019). The genetic instability of AAB is probably attributable to the complexity of genome organization. Some examples reported earlier in the literature have shown the presence of more numerous mobile genetic elements and of duplicated sequences than in ordinary bacteria (Azuma et al., 2009; Zhang et al., 2017; Ryngajłło et al., 2019).

The *Komagataeibacter medellinensis* NBRC 3288 strain (formerly, *Gluconacetobacter xylinus* NBRC 3288) was isolated from rice vinegar as a cellulose-producing strain in 1954, but complete loss of its production capability was reported in 1999 (Navarro and Komagata, 1999). Then, Matsutani et al. (2015) found crucially important gene truncation in the cellulose biosynthesis operon (*bcs* operon) of this strain in 2015. In *Komagataeibacter* bacteria, the *bcs* operon is typically organized as *bcsZHABCD-bglX* or *bcsABXYC*. The genes in these operons encode cyclic dimeric (3′ → 5′) guanosine monophosphate (c-di-GMP) -regulated cellulose synthase (BcsA and BcsB; inner membrane-associated), an outer membrane porin protein (BcsC), putative periplasmic protein (BcsD), cellulose-complementing protein A (BcsH), probable cellulose deacylase (BcsX), probable cellulose acylase (BcsY), endo-β-1,4-glucanase (BcsZ), and β-glucosidase (bglX). Moreover, the genes play a central role in cellulose biosynthesis (Römling and Galperin, 2015).

To elucidate the genomic characterization of nata-de-coco producing bacteria, we determined the whole genome sequences of three bacterial strains (FNDCR1, FNDCF1, FNDCR2) that had been isolated from a practical culture of nata-de-coco in Japan. This report is the first of a study of whole genome sequences of practical nata-de-coco producing bacteria. All of the strains were bacteria of *Komagataeibacter* genus. These data support better understanding of the physiology of *Komagataeibacter* bacteria and elucidate the efficient and stable production of nata-de-coco and biocellulose.

## Materials and Methods

### Bacterial Strains and General Techniques

The nata-de-coco producing bacteria were isolated from a nata-de-coco production facility in Japan. The FNDCR1 strain was stocked in 2017 as a strain that had shown good nata-de-coco productivity from those stored in a nata-de-coco production facility in Japan. The FNDCF1 strain was isolated in 2016 when the productivity loss occurred in the facility. FNDCR2 is a strain that was isolated in 2017 after 20 passages of culture from a stock solution stored at the facility for producing nata-de-coco. In this study, all strains were single colony isolated in 2020, followed by cellulose productivity experiments and whole genome sequencing. Unless stated otherwise, cells were cultured at 28°C using Hestrin–Schramm (HS) medium: 5 g/L yeast extract, 5 g/L peptone, 2.7 g/L Na_2_HPO_4_, 1.5 g/L citric acid, 0.1% (v/v) acetic acid, and 10 g/L sucrose or glucose. For preparation of HS-agar plates, agarose was added at 20 g/L.

### Cellulose Production and Quantification

After fermentation, the cellulose amount was quantified according to the method described in an earlier report, with modifications (Ryngajłło et al., 2019). In brief, cells were spread onto an agar plate (in a 90-mm-diameter Petri dish) from a glycerol stock. After incubating the plate for 4 days, 2 mL of liquid medium was added onto the surface. The cells were scraped and collected. After the optical density of the cell suspension at 600 nm was adjusted to 0.2, 500 μL of the suspension was added to an Erlenmeyer flask containing 9.5 mL of fresh medium. The culture was incubated for 5 days without shaking. The cellulose pellicle was removed from the flask, treated with 1 M NaOH solution overnight in a Petri dish, and washed extensively with distilled water until the water pH became neutral. The washed cellulose was moved to a new and clean Petri dish, of which the weight was pre-recorded. The cellulose was dried completely, typically for 4 h, at 60°C. The Petri-dish and cellulose total weight was recorded.

### Whole-Genome Sequencing

Whole-genome DNA was isolated using the neutral phenol—chloroform—isoamyl alcohol method (Nakashima and Tamura, 2018) with one modification: cellulase (Cellulase, from *Aspergillus niger*; Fujifilm Wako Pure Chemical Corp., Osaka, Japan) was added at 1 mg/mL and incubated for 1 h at 28°C before harvesting. The purified DNA was subjected to sequencing with PacBio RS II and Illumina Hiseq 2,500 systems (Hokkaido System Science Co., Ltd., Sapporo, Japan). The Hiseq reads were obtained with paired-end 100 base pairs (bp) sequencing. The numbers of reads over ≥ Q30 were 47,239,534, 47,458,318, and 44,626,206 bp, respectively, for FNDCR1, FNDCF1, and FNDR2 strains. In the PacBio reads, the numbers of subreads and mean subread lengths were 145,440 and 6,655 bp, 149,926 and 7,350 bp, and 175,664 and 9,362 bp, respectively, for FNDCR1, FNDCF1, and FNDR2 strains. The raw sequencing reads have been deposited in NCBI SRA under their accession numbers SRR15903111-SRR15903116.

The Hiseq reads were trimmed before assembly (TrimGalore! v 0.4.4).^1^ Both the trimmed Hiseq and PacBio reads were used to construct the genomes using the Unicycler software (v. 0.4.6; Wick et al., 2017) with default parameters. The assembly genome completeness was assessed using BUSCO (v.5.2.2; rhodospirillales_odb10) (Manni et al., 2021). The assembled genomes were classified into plasmids and chromosomes using PlasFlow (v. 1.1.0; Krawczyk et al., 2018) with default parameters, respectively. Genome annotation was performed using the prokka (v1.13.7) software (Seemann, 2014) and web-based RAST (v.2.0; Aziz et al., 2008)^2^ with preset parameters, respectively. The CGview server beta was used to illustrate the genome maps^3^ (Grant and Stothard, 2008; Grant et al., 2012). The assembled sequences were deposited in the NCBI Genbank under the BioProject number PRJNA763499.

### Phylogenetic Analysis

From the assembled genomes, 16S rRNA gene sequences were extracted (barrnap v.0.9).^4^ Phylogenetic classification was then performed from the 16S rRNA gene sequences using the Alignment, Classification and Tree Service^5^ of SILVA (Release 138; Quast et al., 2013).

Based on the classification results, the public genome of an assigned genus (*Komagataeibacter* genus) was obtained from NCBI Genbank^6^ using NCBI-genome-download v.0.3.0.^7^ To ensure the genome assembly levels, the downloaded genomes were limited to “complete” whole genomes.

To identify the core gene sets from three new assembly genomes and downloaded genomes, a pan-genomic analysis was performed (Roary v.3.11.2; Page et al., 2015). Using these core gene sets, a maximum-likelihood phylogenetic tree was constructed with IQ-TREE v.2.0.3 (bootstrap: 1,000 replicates; best-fitting substitution model: GTR + F + R6 model) (Nguyen et al., 2015). The phylogenetic tree was visualized using iTOL (v.6.3)^8^ (Letunic and Bork, 2021).

### Identification of Synteny Blocks and Duplicated Regions

To detect synteny blocks, the three assembled genomes were subjected to pairwise comparisons to themselves and other genomes (Sibelia v.3.0.7; Minkin and Medvedev, 2020). The detected synteny blocks were visualized as paths between genomes (Circa v.1.2.2; OMGenomics). Duplicate regions within the genome were sought (RepSeek v.6.6; Achaz et al., 2006) with minimum length thresholds of 1,000, 2,000, and 3,000 bp. Moreover, correlation tests and linear regression analyses were conducted using implemented functions in R language (R v.4.1.1; R Development Team, 2021) to assess the relation between the duplicated lengths and the number of mobile genetic elements. The R script are available on https://github.com/omics-tools/natagenome_rscript.

## Results

### Validation of Cellulose Productivity

**FIGURE 1:**
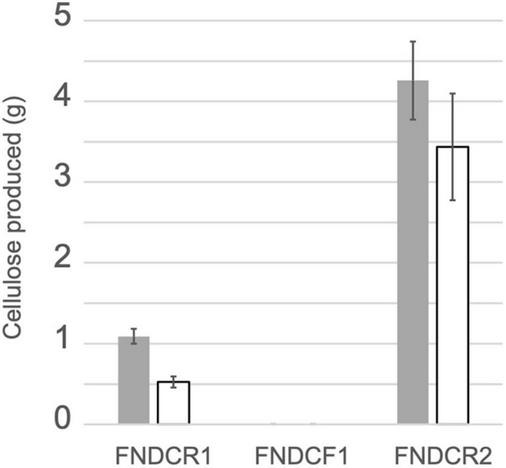
Cellulose productivity of the present strains. Cellulose was produced from glucose (gray bars) or sucrose (open bars) using the designated strains. Quantification results of cellulose from three replicate experiments are shown as mean ± SEM.

Cellulose productivity of three strains was quantified as described in section “Materials and Methods” (Figure 1). As carbon sources, glucose or sucrose was fed to the cells. The FNDCR2 strain produced more cellulose than the FNDCR1 strain, although the FNDCF1 strain did not produce at all. This result indicates that the nata-de-coco producing bacterial culture contained both cellulose producing and non-producing cells.

### Whole Genome Sequence Determination

**FIGURE 2:**
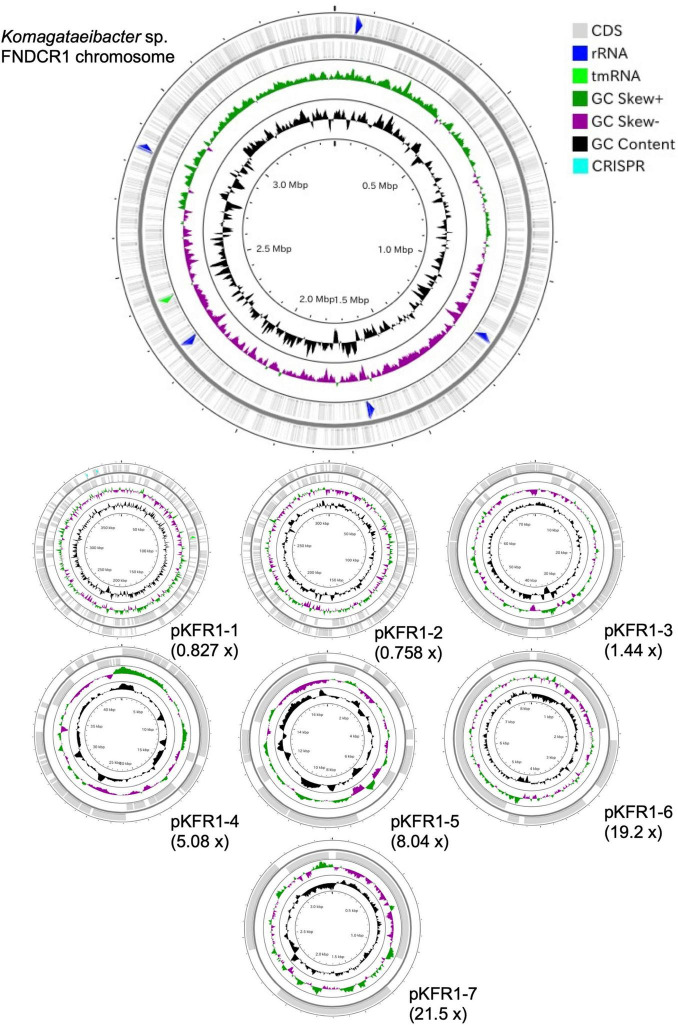
Genome structure of the FNDCF1 strain. One circular chromosome and seven putative plasmids (pKFR1-1, pKFR1-2, pKFR1-3, pKFR1-4, pKFR1-5, pKFR1-6, and pKFR1-7) are shown. The value presented in parentheses represents the sequencing coverage of a plasmid relative to that of chromosome. Smaller contigs below 5,000 bp and non-circularized contigs are not shown in this figure.

**FIGURE 3:**
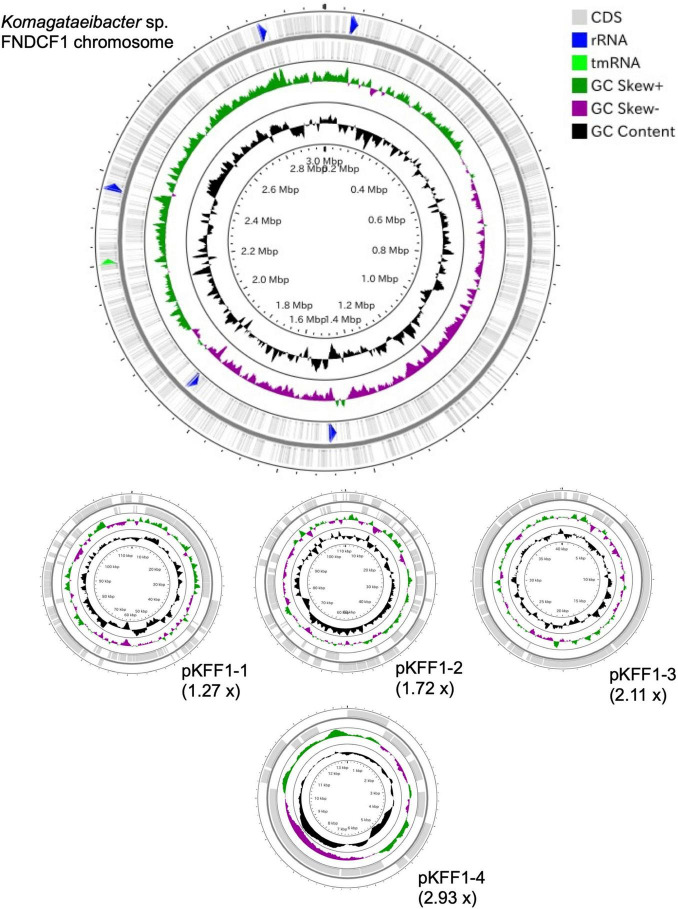
Genome structure of the FNDCR1 strain. One circular chromosome and four putative plasmids (pKFF1-1, pKFF1-2, pKFF1-3, and pKFF1-4 pKFR1-7) are shown. The value given in parentheses represents the sequencing coverage of a plasmid relative to that of chromosome. Smaller contigs below 5,000 bp and non-circularized contigs are not shown in this figure.

**FIGURE 4:**
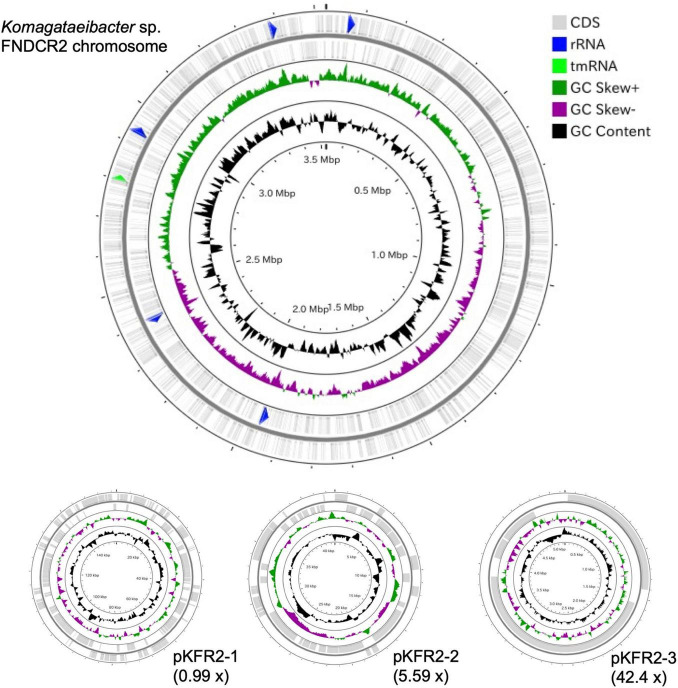
Genome structure of the FNDCR2 strain. One circular chromosome and three putative plasmids (pKFR2-1, pKFR2-2, pKFR2-3) are shown. The value given in parentheses represents the sequencing coverage of a plasmid relative to that of chromosome. Smaller contigs below 5,000 bp and non-circularized contigs are not shown in this figure.

To identify the taxonomy and to investigate cellulose biosynthesis mechanisms of the present strains, the genome sequences were obtained with shotgun sequencing. For constructing complete genome sequences, both short-read and long-read sequencing were performed. After sequencing, the whole genome sequences were constructed with hybrid assembly (section “Materials and Methods”). The assembled genomes of FNDCR1, FNDCF1, and FNDCR2, respectively, showed high completeness of 99.5, 99.6, and 99.7%. All the strains were found to harbor multiple circular plasmids in addition to circular chromosomes (Figures 2–4). There was no shared plasmid in these three strains (Supplementary Figure 1). The relative value of sequencing coverage of each plasmid is expected to reflect the plasmid copy number. Two CRISPR sequences were found on the pKFR1-1 plasmid.

### Phylogenetic Relation Analysis

**FIGURE 5:**
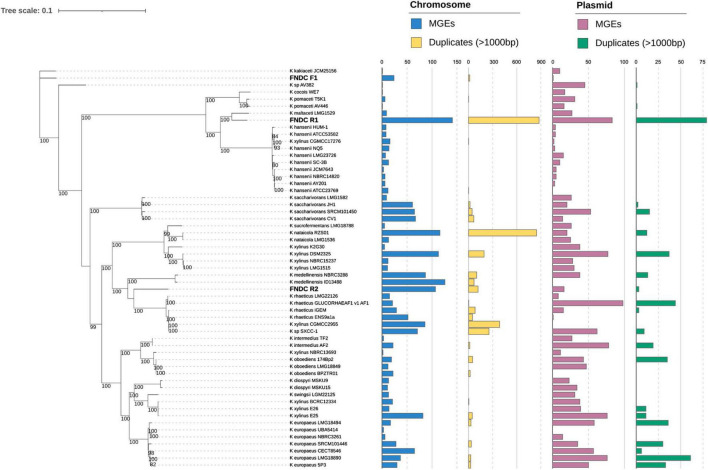
Maximum-likelihood phylogenetic tree in *Komagataeibacter* genus. A maximum-likelihood phylogenetic tree was built using IQ-TREE based on the public *Komagataeibacter* genomes and the present strain genomes (FNDCR1, FNDCF1, and FNDCR2). The numbers at nodes are the bootstrap values > 80% as a percentage of 1,000 replications. The bar plot on the right shows the number of mobile genetic elements (MGEs) and Duplicates > 1,000 bp, respectively, on the chromosome and plasmid. The term “Duplicates” refers to homologous sequence pairs in the genome, as detected by RepSeek. MGEs and Duplicates on chromosomes, respectively, correspond to cyan and yellow, whereas MGEs and Duplicates on plasmids, respectively, correspond to pink and green.

Each strain harbored five 16 rRNA genes on the chromosome. All 16S rRNA sequences of the FNDCR1, FNDCF1, and FNDCR2 strains were assigned to those of *Komagataeibacter* with > 99% similarities (Supplementary Table 1). The phylogenetic tree constructed based on the core gene set derived from the pan-genome analysis of *Komagateibacter* (Figure 5) shows that the non-cellulose synthesizing type, FNDCF1, is in a clade close to the root in the phylogenetic tree of *Komagateibacter* bacteria. This strain is close to the *Komagataeibacter kakiaceti* JCM25156 strain isolated from traditional Japanese fruit vinegar (Iino et al., 2012). The cellulose-producing type FNDCR1 is located close to the vinegar-producing acetic acid bacterium *Komagataeibacter maltaceti* LMG 1529 (Zhang et al., 2018). The cellulose-producing type FNDCR2 is represented as a single clade, with the larger clade being closer to the *Komagataeibacter rhaeticus* lineage. However, none of the present genomes is the same as known strain genomes. These results indicate that all the present strains belong to *Komagataeibacter* genus, but all might be novel strain genomes.

### Analysis of Genome Structure and Genome Complexity in Cellulose Producing Strains

**FIGURE 6:**
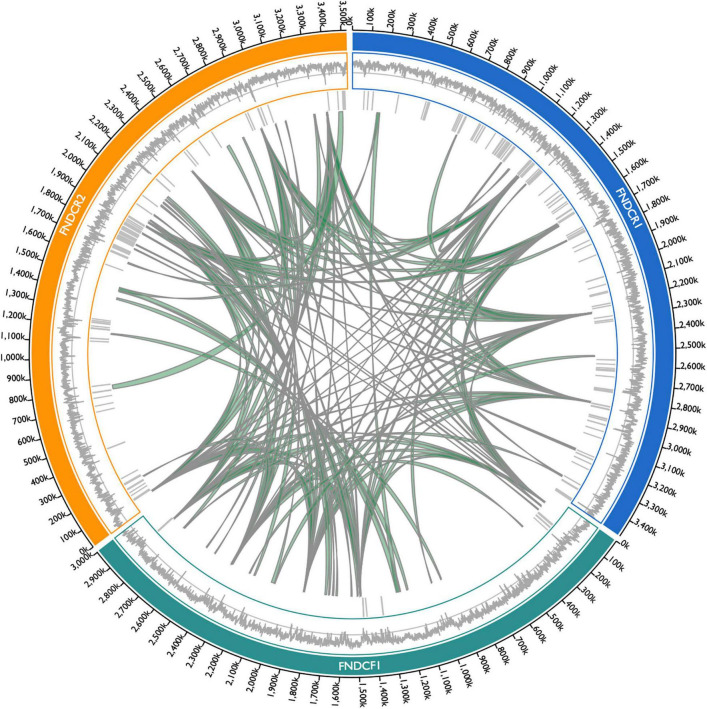
Synteny blocks among the present strain chromosomes. Synteny blocks > 5,000 bp within/among chromosome genomes of FNDCR1 (cyan), FNDCF1 (light green), and FNDCR2 (orange) are shown as gray-colored paths. Synteny blocks were detected using Siberia. The barcode plot outside the synteny block shows where the MGEs are located in the genome. The outer line plot shows the GC content in the genome. The middle line shows the 50% GC content. Numbers on the outside represent the chromosome positions.

**FIGURE 7:**
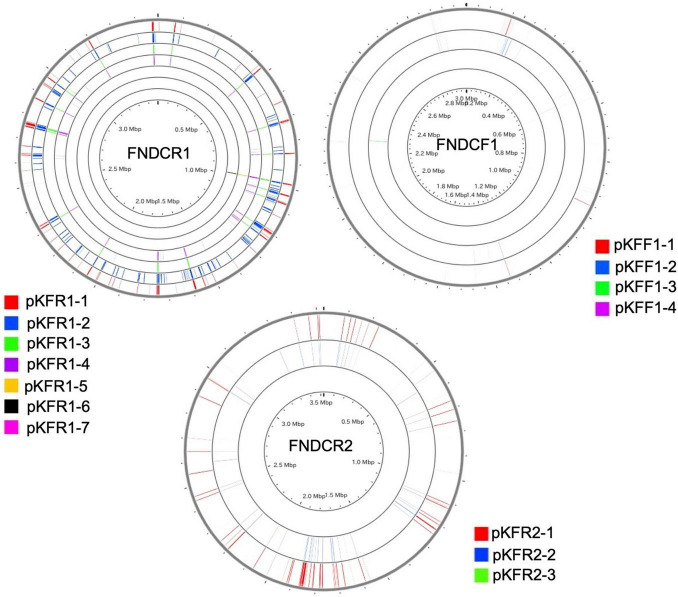
Sequences shared with the chromosome and the plasmids. Homology search (blast algorithm) between each chromosome and plasmids in each strain (other circles) was performed on the CGview server beta. Each plasmid sequence was used as a query. Homologous sequences found on the chromosome are marked with colored lines.

To compare the genome structures of the respective strains, the three assembled genomes were searched for synteny blocks between themselves and other genomes. Although homologous synteny blocks were found between the three genomes, the overall genome structure did not closely match each other (Figure 6). Results show that the plasmids in each strain harbor regions with DNA homologous to the chromosome (Figure 7). For the plasmids in the FNDCR1 and FNDCR2 strains, many homologous regions were found in their chromosomes, although few such regions were found in the FNDCF1 chromosome.

**FIGURE 8:**
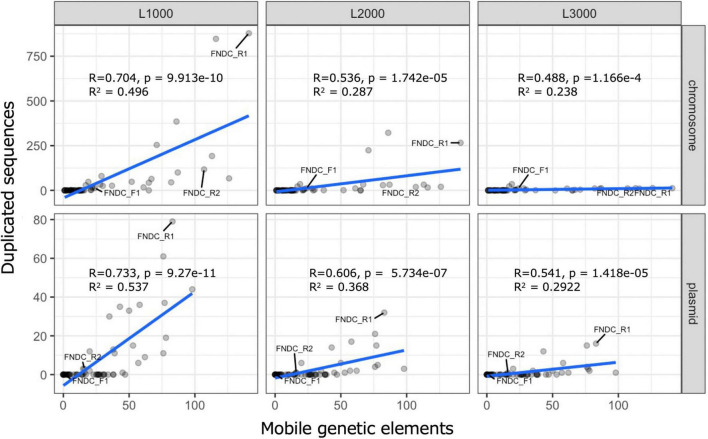
Scatter plots for duplicated sequences and mobile genetic elements. These scatter plots show results of correlation and regression analysis of the number of duplicated sequences and mobile genetic elements in the genome. Correlation analysis and regression analysis were performed with functions implemented in R. The top and bottom rows, respectively, present results for chromosomes and plasmids. Columns L1000, L2000, and L3000, respectively, correspond to the conditions that set 1,000, 2,000, and 3,000 bp as the minimum length threshold during the duplicate sequence search. The line written in blue is the regression line. *R*, *p*, and *R*^2^, respectively, denote the correlation coefficient, the *p*-value of the uncorrelation test, and the multiple correlation coefficient.

**FIGURE 9:**
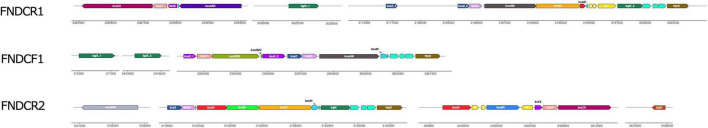
Cellulose biosynthesis operons of the present strains. The figure portrays the operon structure for genes involved in cellulose biosynthesis. The structure was drawn based on results obtained for gene annotation (Supplementary Table 1). Rectangles with arrowheads represent regions that encode genes, open reading frames (ORFs), or transposons (ISs). The arrowhead direction shows the DNA strand orientation (forward/reverse). The numbers represent the genomic positions in the chromosomes. Colors correspond to the encoded genes or ISs. Those without names are the predicted ORFs.

For the whole genomes in the FNDCR1 and FNDCR2 strains, many duplicated sequences were observed, but these characteristic structures were not found in FNDCF1 strain (Figures 5, 8). Furthermore, to investigate relationships between these duplications and mobile genetic elements, the comparative genome analysis was performed with published other *Komagataeibacter* genomes. Detected duplicates with minimum length of 1,000 bp were significantly and positively correlated with the number of mobile genetic elements in the genome. This relation was more apparent for plasmids than for chromosomes (Figure 8). However, for duplicates with 2,000 bp or greater length, no positive correlation was found either for chromosomes or for plasmids. These results suggest that mobile genetic elements can be associated with the transposition of sequences less than 2,000 bp on their genomes. In addition, the assembled genome of FNDCR1 clearly has more duplicated sequences than the other strains, suggesting the existence of a strain-specific evolutionary event related with accelerated sequence duplication. The coding sequences on these repeats and their neighbors have been summarized as Supplementary Table 2 and Supplementary Figure 2. As a result, about 30 and 19% of the repeated coding sequences in chromosomes and plasmids were annotated as genes related to mobile genetic elements, respectively (Supplementary Figure 2). This result supports the association between the number of mobile genetic elements in the genome and the number of repeats as shown in Figures 5, 8. Most of the other repeated coding sequences were annotated as hypothetical proteins, and their functions and effects are not well understood. Interestingly, some cellulose synthesis-related genes (cellulose synthase 1, cellulose synthase operon protein C and D) were also observed in the repeated and repeated-neighbor regions in chromosomes (Supplementary Table 2). Transposons (ISs) were also observed in the neighboring region of the cellulose synthesis operon of the three strains in this study (Figure 9). These results suggest that mobile genetic elements in *Komagataeibacter* genomes may also affect the loss and gains of cellulose synthesis-related genes.

The sequence of each plasmid was compared to those of all other plasmids presented herein, as well as those of plasmids found previously in *K. xylinus* E25 strain (Supplementary Figure 1). Actually, pKFR1-2, pKFR2-1, and pGX5 (*K. xylinus* E25) were mutually similar. Also, pKFR1-4 was similar to pKFR2-2 throughout the plasmids. Moreover, pKFF1-4, pKFR1-4, pKFR2-1, pGX4 (*K. xylinus* E25), and pGX3 (*K. xylinus* E25) shared partial homology: an approximately 3 kb part of pKFR1-7 (total length of 3.4 kb) was highly homologous to the part of pKFR1-6 and pGX1 (*K. xylinus* E25). In addition, a consensus sequence of approximately 3.7 kb in pKFR1-1, pKFR1-2, pKFR1-3, pKFR1-4, pKFR1-5, pKFR1-6, pKFR2-1, and pKFR2-2 was identified. On this sequence, only transposase and resolvase were coded. Therefore, the same transposable elements were present on these plasmids.

Taking these findings together, one can infer that the genome structure of FNDCR1 and FNDCR2 strains are complex, leading us to predict that the genomes of these strains are genetically unstable.

### Structure of the Cellulose Biosynthesis Operon

For cellulose biosynthesis, the *bcsABCD* genes are the most important genes: with other accessory genes, they are involved in efficient biosynthesis (Römling and Galperin, 2015). We manually extracted information related to *bcs* and other genes from the gff3 annotation file created using the prokka and RAST programs (Figure 9). It is noteworthy that many strains of *Komagataeibacte*r bacteria harbor two *bcs* operons (type I and II). Typically, the type I and II operons, respectively, comprise *bcsZ-bcsH-bcsAI-bcsBI-bcsCI-bcsDI-bglX*, and *bcsAII-bcsBII-bcsX-bcsY-bcsCII* (the *bcsA* and *bcsB* genes are sometimes found as a fused gene, *bcsAB*) (Römling and Galperin, 2015; Ryngajłło et al., 2019).

The FNDCR1 and FNDCR2 strains harbored operons of both types as intact, however, only one *bcs* operon was found in the FNDCF1 strain. The sole *bcs* operon of the FNDCF1 strain was a fused one of type I and II operons, respectively. Furthermore, no *bcsCI*, *bcsAII*, or *bcsBII* gene was found. A premature stop codon appeared in the C-terminal part of *bcsCII* in the FNDCF1 strain (Figure 9). The loss of these genes can have affected the function of guanosine monophosphate (c-di-GMP)-regulated cellulose synthase and outer membrane porin proteins in the FNDCF1 strain. This operon structure explains why this strain does not produce cellulose at all.

We also found that the genes for Tiorf138 protein, hypothetical protein of Cupin superfamily, hydrolase of NUDIX family, and chaperone protein HtpG were located immediately downstream of *bcsDI* in all the presented strains. These four genes were also located similarly on the chromosomes of *Komagataeibacter* spp. E25, NBRC3288, and ATCC23769 strains (Supplementary Table 3), implying possible involvement of these genes in cellulose biosynthesis. The “Discussion” section provides additional details.

## Discussion

The genetic instability of *Komagataeibacter* bacteria at least partly derives from the presence of the exceptionally huge number of mobile genetic elements on the genomes (Azuma et al., 2009; Zhang et al., 2017). Furthermore, high degrees of gene duplication, hyper-mutability, and complex plasmid contents have been reported for many *Komagataeibacter* bacteria (Römling and Galperin, 2015). The present study revealed a similar complex genomic structure of nata-de-coco producing *Komagataeibacter* bacteria. These findings show many truncations or insertions of ISs within the *bcs* operon genes of the present and other *Komagataeibacter* strains (Supplementary Table 3). These genetic variations are inferred to be the result of genomic structure complexity. Additionally, this report is the first of a positive correlation found between the number of mobile genetic elements and the number of duplicated sequences on the genomes (Figure 8) and the first describing that not all genomes of *Komagataeibacter* strains are complex (Figures 7, 8; the FNDCF1 strain had the simple genome).

The *bcsCII* gene of the FNDCF1 strain had a premature stop codon. Some *bcs* genes were not found on the genome (Figure 9). In the *Komagataeibacter medellibensis* NBRC3288 strain, the loss and restoration of cellulose productivity were observed according to a loss-of-function mutation (frameshift mutation) and intragenic suppressor mutations (reversion of frameshift) in the *bcsBI* gene (Matsutani et al., 2015). Furthermore, *Komagateeibacter europaeus* LMG18890 and LMG18494 strains are non-cellulose producers. They have some impairments in either *bcsBI* or *bcsCI* genes (Matsutani et al., 2015). A non-sense mutation in the *bcsCI* gene of *Komagataeibacter oboediens* MSKU3 has been reported to engender loss of cellulose productivity (Taweecheep et al., 2019). Therefore, we infer that the loss of cellulose productivity in the FNDCF1 strain is attributed to loss of a full functional set of the *bcs* operon.

Expression from the *bcs* operon in *Komagataeibacter* bacteria is known to be highly stimulated by c-di-GMP (Zhang et al., 2017). Therefore, the intracellular level of c-di-GMP is important for cellulose production. However, the FNDCF1 strain harbored five intact diguanylate cyclase/phosphodiesterase genes that are necessary to synthesize c-di-GMP, suggesting that the c-di-GMP level is normal in this strain. Similarly, the FNDCR1 and FNDCR2 strains, respectively, harbored five and two diguanylate cyclase/phosphodiesterase genes.

The genes for Tiorf138 protein, hypothetical protein of Cupin superfamily, hydrolase of NUDIX family, and chaperone protein HtpG are located immediately downstream of the *bcs* operon in many *Komagataeibacter* strains (Figure 9 and Supplementary Table 3). Hydrolases of NUDIX (nucleoside diphosphates-linked moiety-X) family hydrolyzes the pyrophosphate linkage in various nucleoside diphosphates linked to another moiety X (NDP-X) such as pyridine nucleotides, coenzyme A, dinucleoside polyphosphates, and nucleotide sugars (Fisher et al., 2002). Uridine-5′-diphosphate-α-D-glucose (UDP-glucose), a substrate for cellulose polymerization in bacteria (Ryngajłło et al., 2019), is a kind of NDP-X (a nucleotide sugar). In the case of glycogen biosynthesis in which UDP-glucose is a polymerization substrate, NUDIX hydrolases are thought to influence the flux of glucose into glycogen (Heyen et al., 2009). Therefore, the NUDIX hydrolases near the *bcs* operons might be involved in UDP-glucose degradation and might regulate cellulose biosynthesis. In this respect, determining the substrate specificity of those NUDIX hydrolases is important. Actually, HtpG, a chaperon protein of hsp90 family, is known to interact with proteins containing tetratrico peptide repeat (TPR) domain (Scheufler et al., 2000). In fact, because the N-terminal portion of BcsC contains 17 TPR, HtpG might interact with BcsC (Du et al., 2016; Nojima et al., 2017). Additionally, in *Pseudomonas aeruginosa*, results demonstrated that low intracellular levels of c-di-GMP increase the intracellular HtpG level (Chua et al., 2013).

In the phylogenetic classification based on 16S rRNA genes, all three isolated strains were assigned to *Komagataeibacter* genus. Phylogenetic tree analysis of the conserved core genes indicated that the three strains belong to mutually different clades. None of them matches any known strain. This finding suggests that the present strains are novel strains that have not been reported in the literature as *Komagataeibacter* strains. Duplicated genome regions as their genomic signature were also specifically examined. In fact, FNDCR1 and FNDCR2 have many duplicated sequences within their own genomes. By contrast, FNDCF1 has only a few duplicates in its own genome. It is particularly interesting that FNDCF1 has far fewer mobile genetic elements in its genome than the other two cellulose-producing lineages (FNDCR1 and FNDCR2), which might be attributable to the cross-strain variation in the sequence duplication among *Komagataeibacter* strains. After applying correlation analysis between the length of these homologous duplicated sequences and the number of mobile genetic elements, duplicates with minimum length of 1,000 bp were found to be positively correlated with the number of mobile genetic elements. Nevertheless, no correlation was found for duplicates with minimum length longer than 2,000 bp. This finding suggests that mobile genetic elements are related with transposition in the genome, but they might be subject to negative selection in their duplicated sequence lengths.

Our initial motivation for this study was to understand the genetic instability of nata-de-coco producing bacteria and stabilization of nata-de-coco production yields. For industrial production of acetic acid, *Acetobacter pasteurianus* 386B strain is exploited frequently. Its low number of mobile genetic elements in the genome and absence of complete phage genomes makes this strain more genetically stable than other *A. pasteurianus* strains (Illeghems et al., 2013; Ryngajłło et al., 2019). From results of the comparative genomics study presented herein, although FNDCF1 lacks the ability to produce cellulose, it can be expected to have less influence of mobile factors on genome stability. Given these findings, after the *bcs* operon of FNDCR1 or FNDCR2 strains is genetically transferred to the chromosome of FNDCF1 strain, it can be anticipated for use in stable industrial nata-de-coco production. To this end, useful genetic engineering technologies including genome editing should be developed for *Komagataeibacter* bacteria.

## Data Availability Statement

The datasets presented in this study can be found in online repositories. The names of the repository/repositories and accession number(s) can be found below: https://www.ncbi.nlm.nih.gov/bioproject/PRJNA763499, PRJNA763499.

## Author Contributions

KI and NN designed the study, collected the data, conducted data analyses, and drafted the manuscript. HK, TI, and KK reviewed the manuscript. All authors read and approved the manuscript.

## Conflict of Interest

HK was employed by company Research and Development Department, Fujicco Co., Ltd. The remaining authors declare that the research was conducted in the absence of any commercial or financial relationships that could be construed as a potential conflict of interest.

## Publisher’s Note

All claims expressed in this article are solely those of the authors and do not necessarily represent those of their affiliated organizations, or those of the publisher, the editors and the reviewers. Any product that may be evaluated in this article, or claim that may be made by its manufacturer, is not guaranteed or endorsed by the publisher.

## Footnotes

1
https://github.com/FelixKrueger/TrimGalore

2
https://rast.nmpdr.org/

3
http://cgview.ca

4
https://github.com/tseemann/barrnap

5
https://www.arb-silva.de/aligner/

6
https://www.ncbi.nlm.nih.gov/genbank/

7
https://github.com/kblin/ncbi-genome-download

8
https://itol.embl.de/
