# Supplementary material for: Comparative Genome Analysis of Three Komagataeibacter Strains Used for Practical Production of Nata-de-Coco
Source: Front Microbiol. 2022 Feb 4;12:798010. doi: 10.3389/fmicb.2021.798010 (PMC8855687; doi:10.3389/fmicb.2021.798010)
Supplement: Supplementary file 1 [file Data_Sheet_1.pdf]

Supplementary Materials

Fig. S1

(A) pKFR1-1

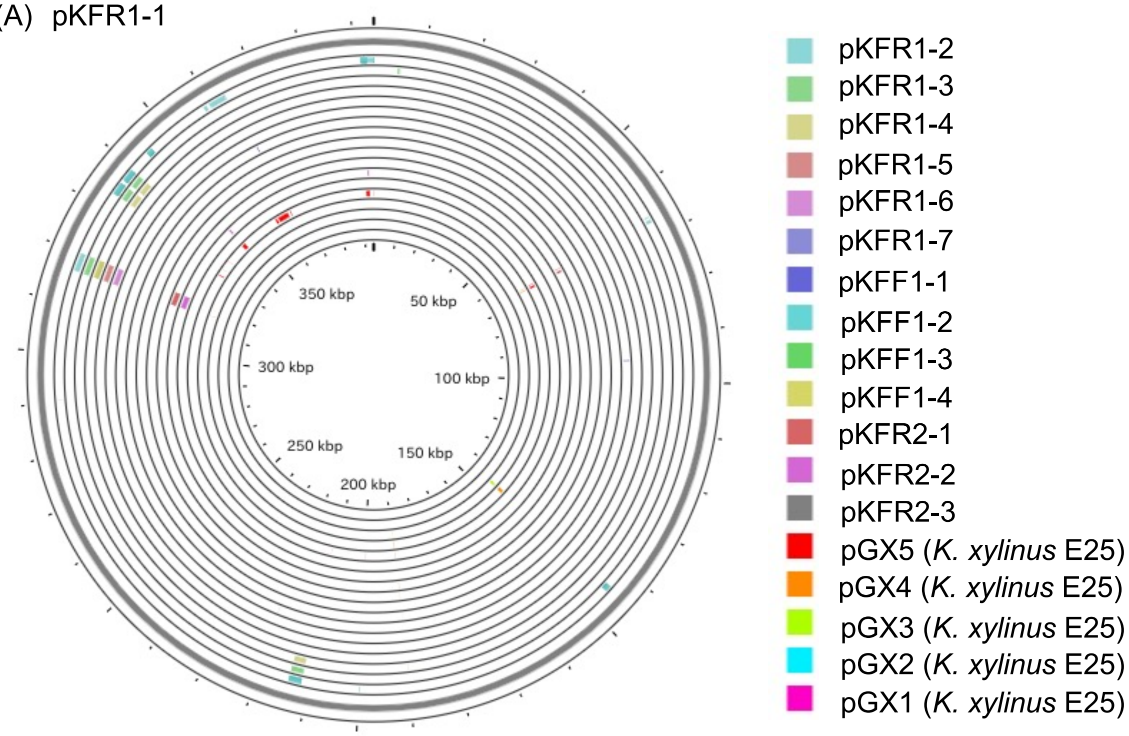

(B) pKFR1-2

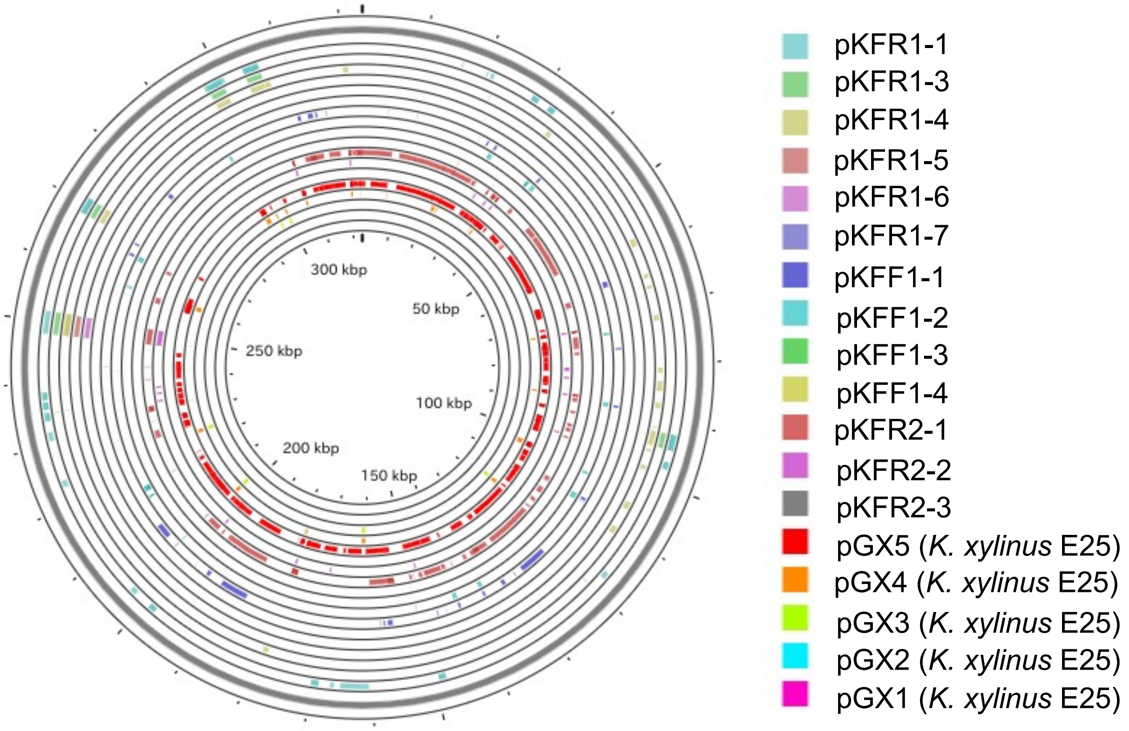

Fig. S1

(C) pKFR1-3

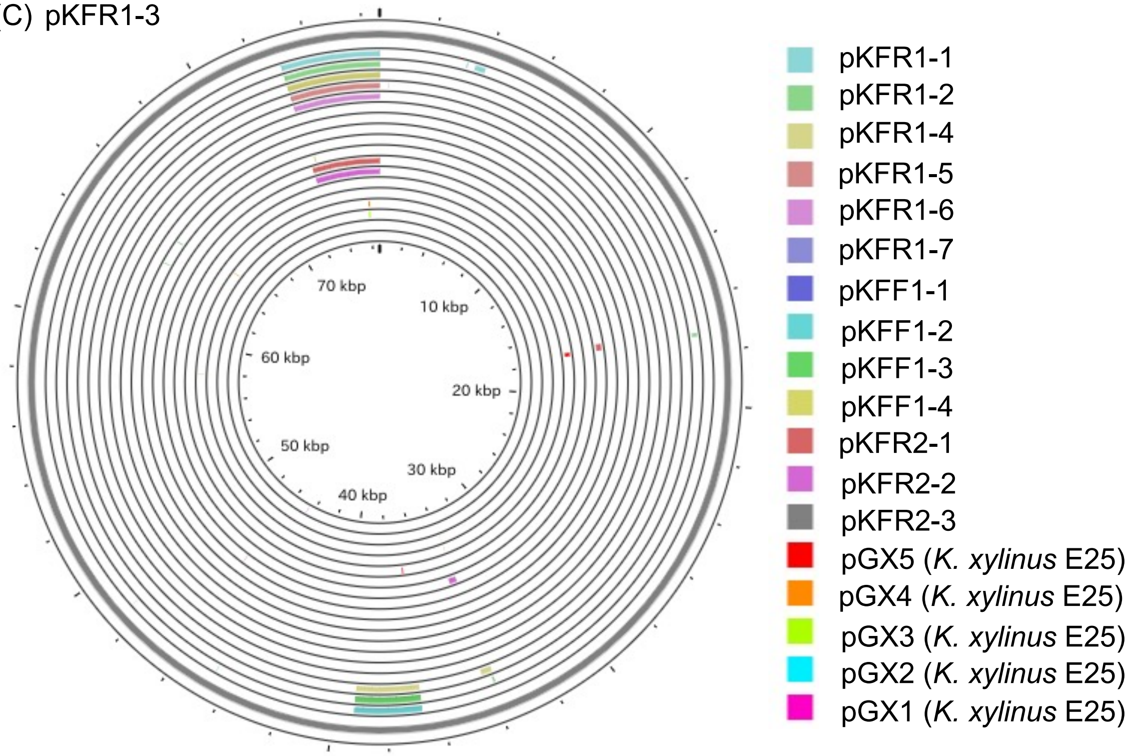

(D) pKFR1-4

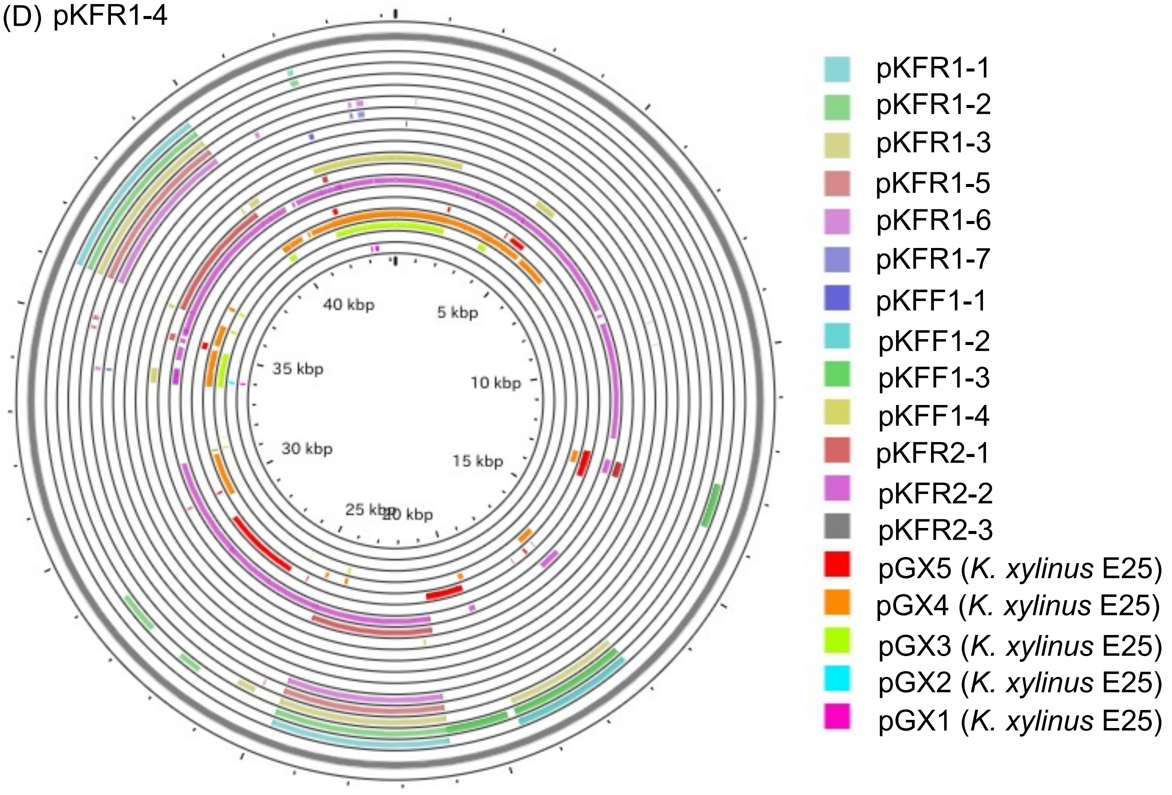

Fig. S1

(E) pKFR1-5

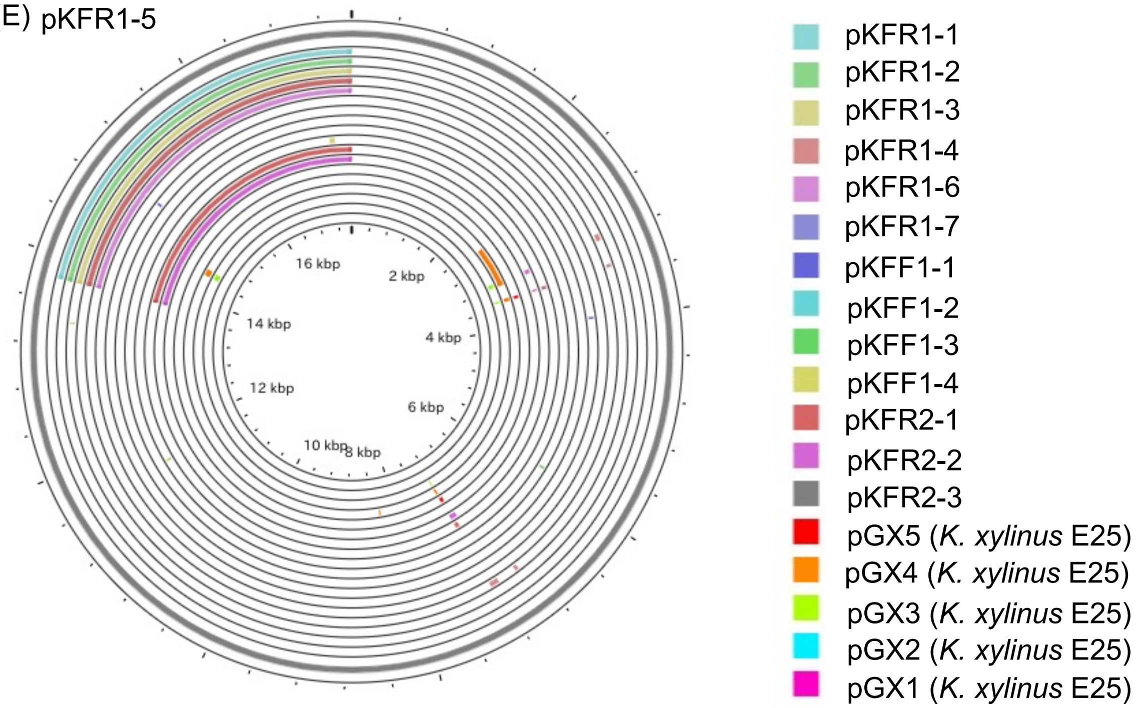

(F) pKFR1-6

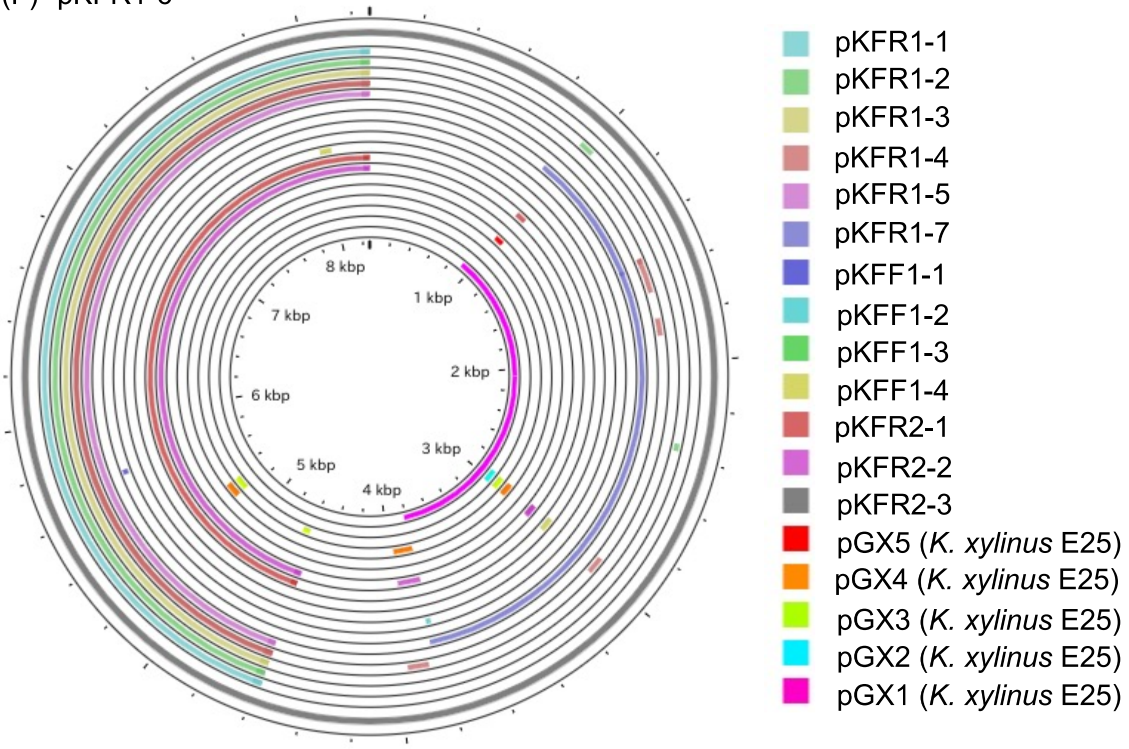

Fig. S1

(G) pKFR1-7

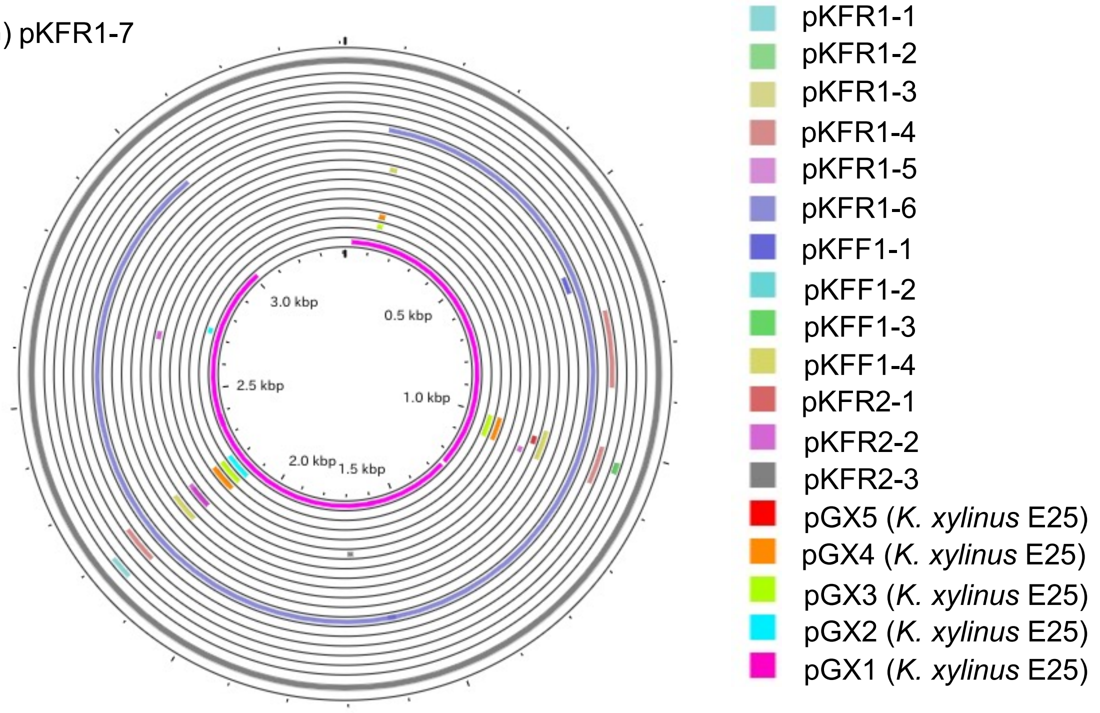

(H) pKFF1-1

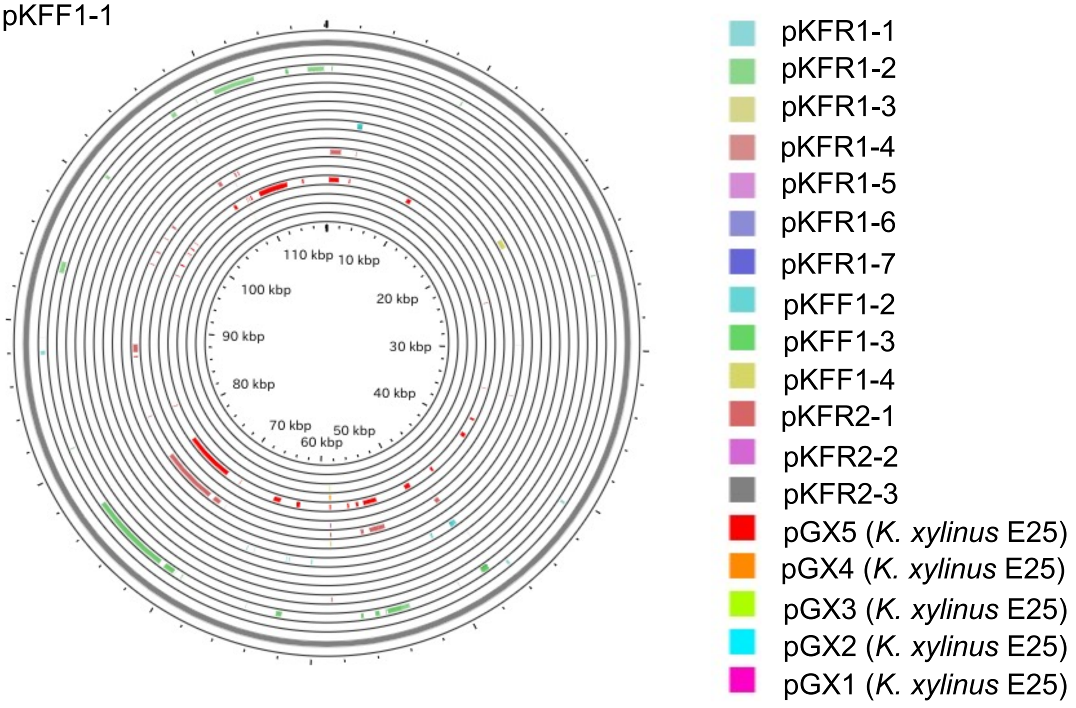

Fig. S1

(I) pKFF1-2

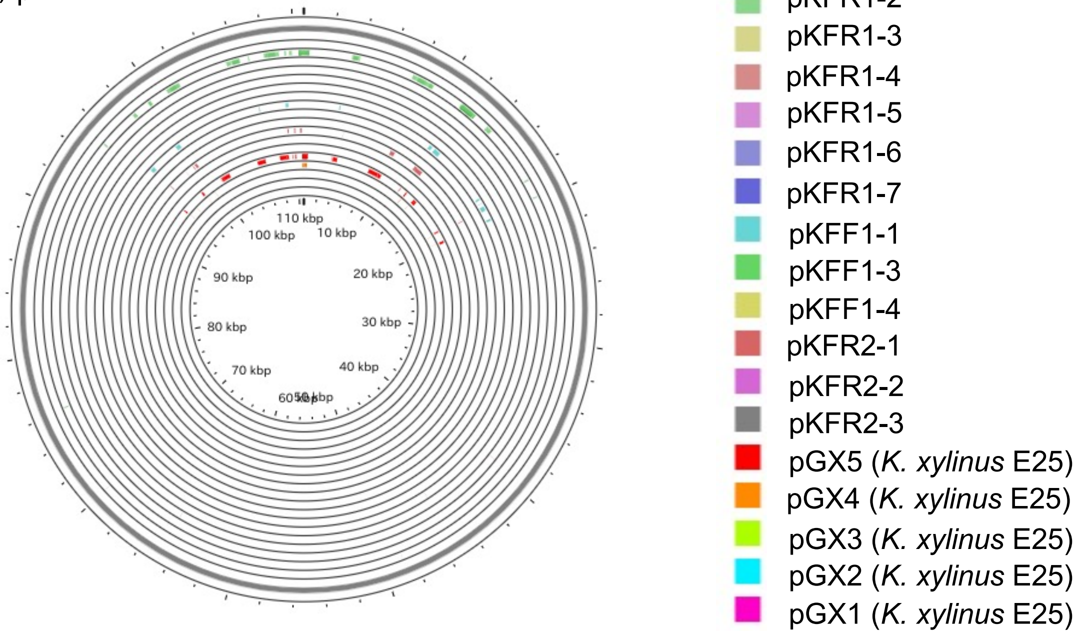

(J) pKFF1-3

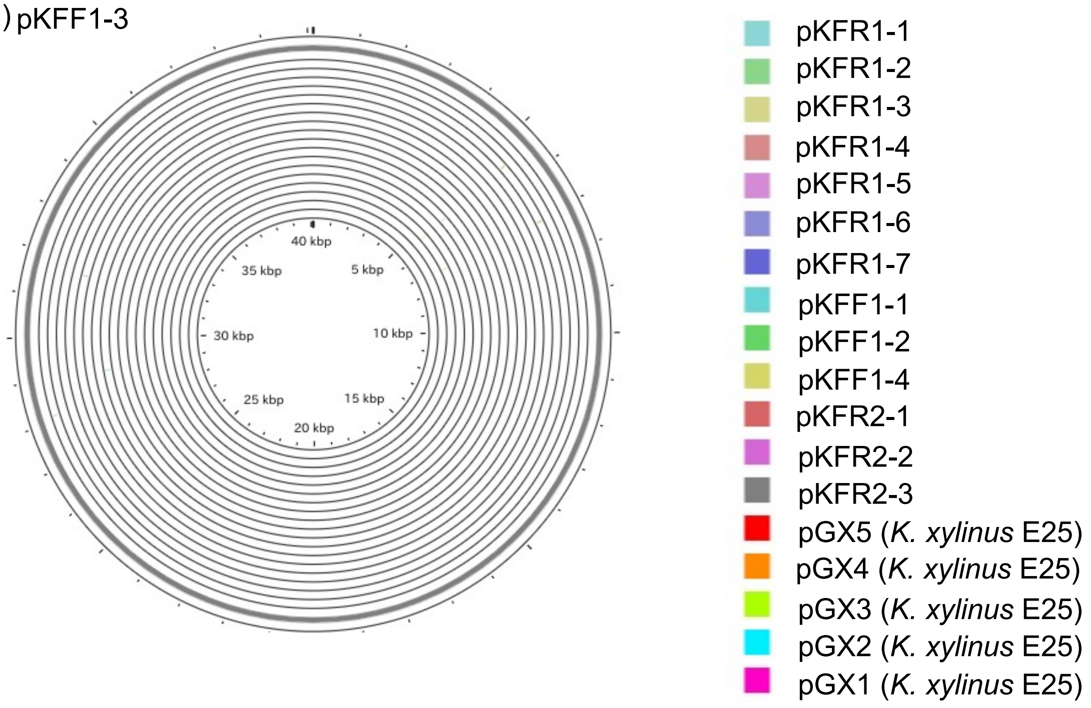

Fig. S1

(K) pKFF1-4

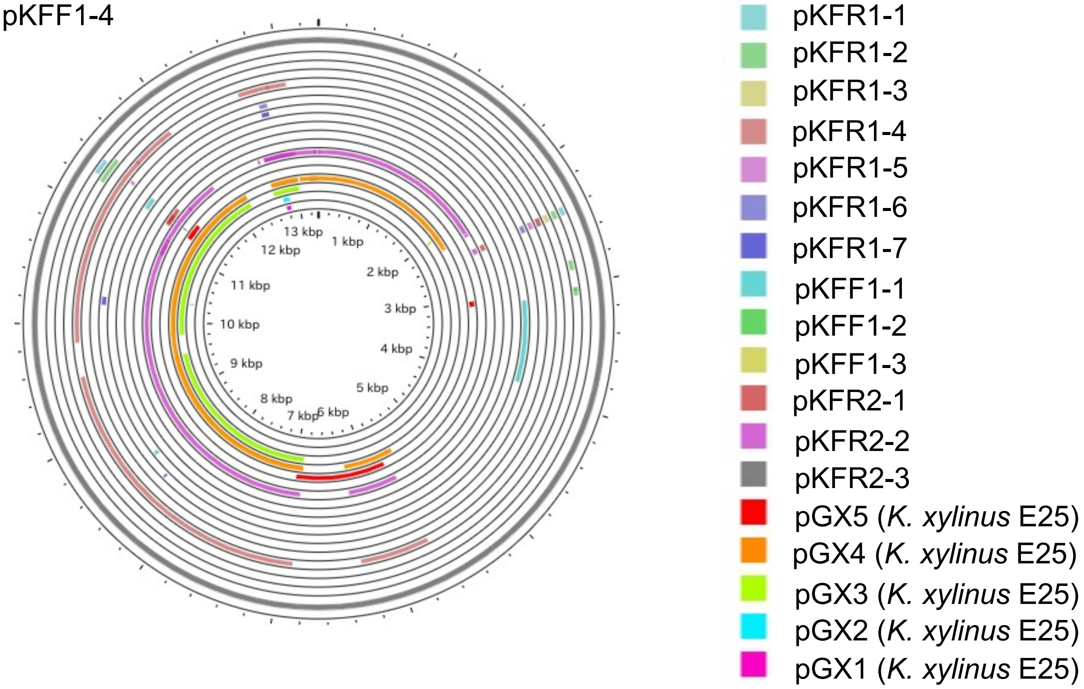

(L) pKFR2-1

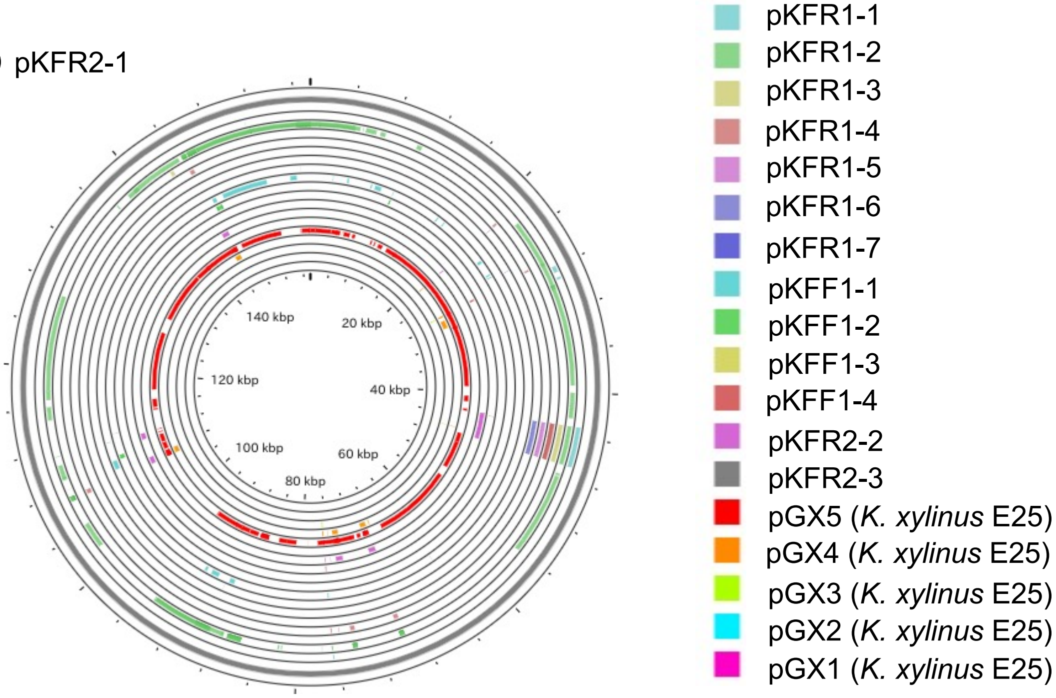

Fig. S1

(M) pKFR2-2

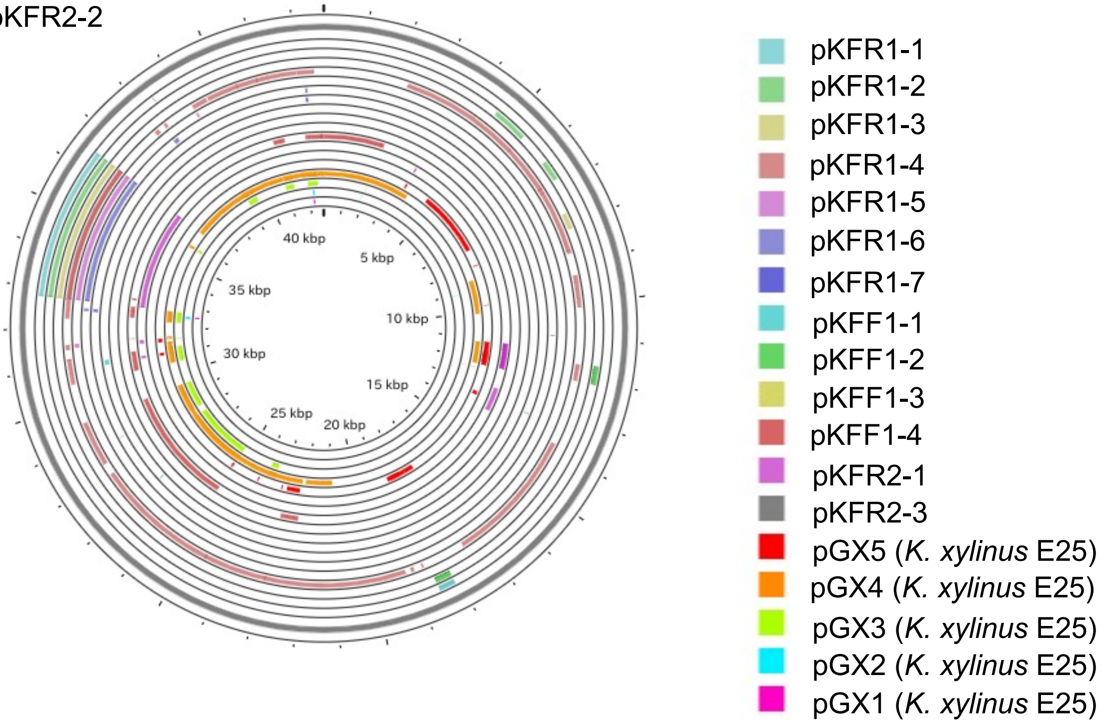

(N) pKFR2-3

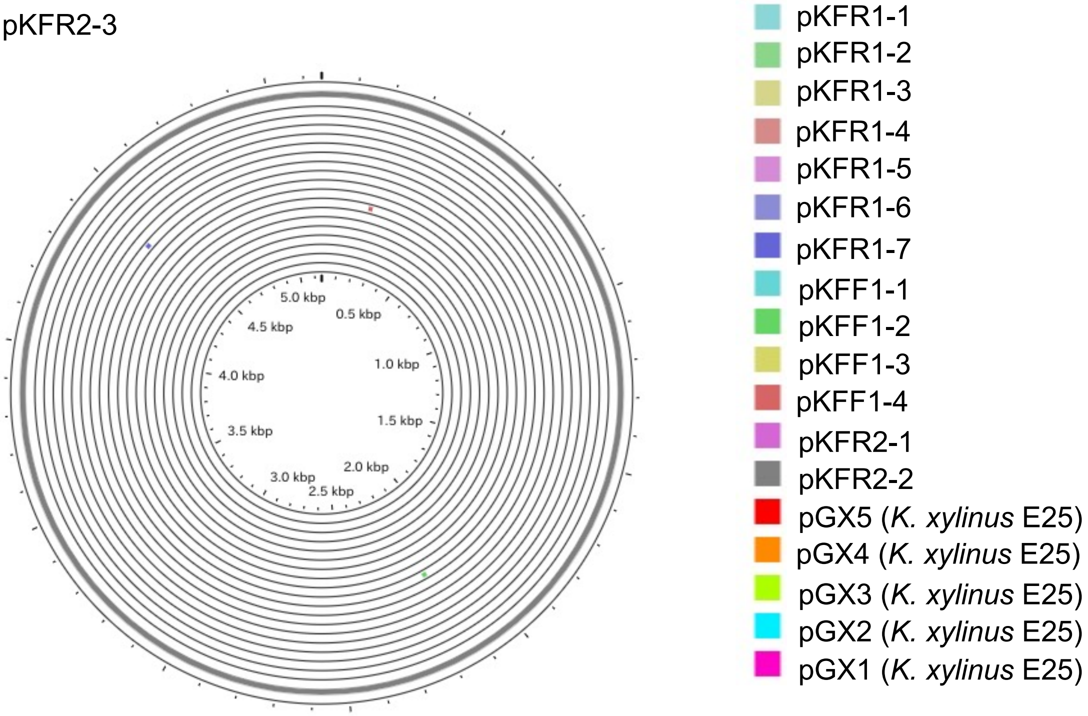

Supplementary Figure S1. Homology among plasmids. Homology search (blast algorithm) between one plasmid (indicated at the upper left in each panel) and other plasmids (indicated right with color guides in each panel) was performed on the CGview server beta [http://cgview.ca/; Nucleic Acids Res (2008) 3:W181–W184.; BMC Genomics (2012) 13:202]. Expect value cutoff was set to 0.1. The plasmids of the K. xylinus E25 strain was reported earlier [MicrobiologyOpen (2018) e731].

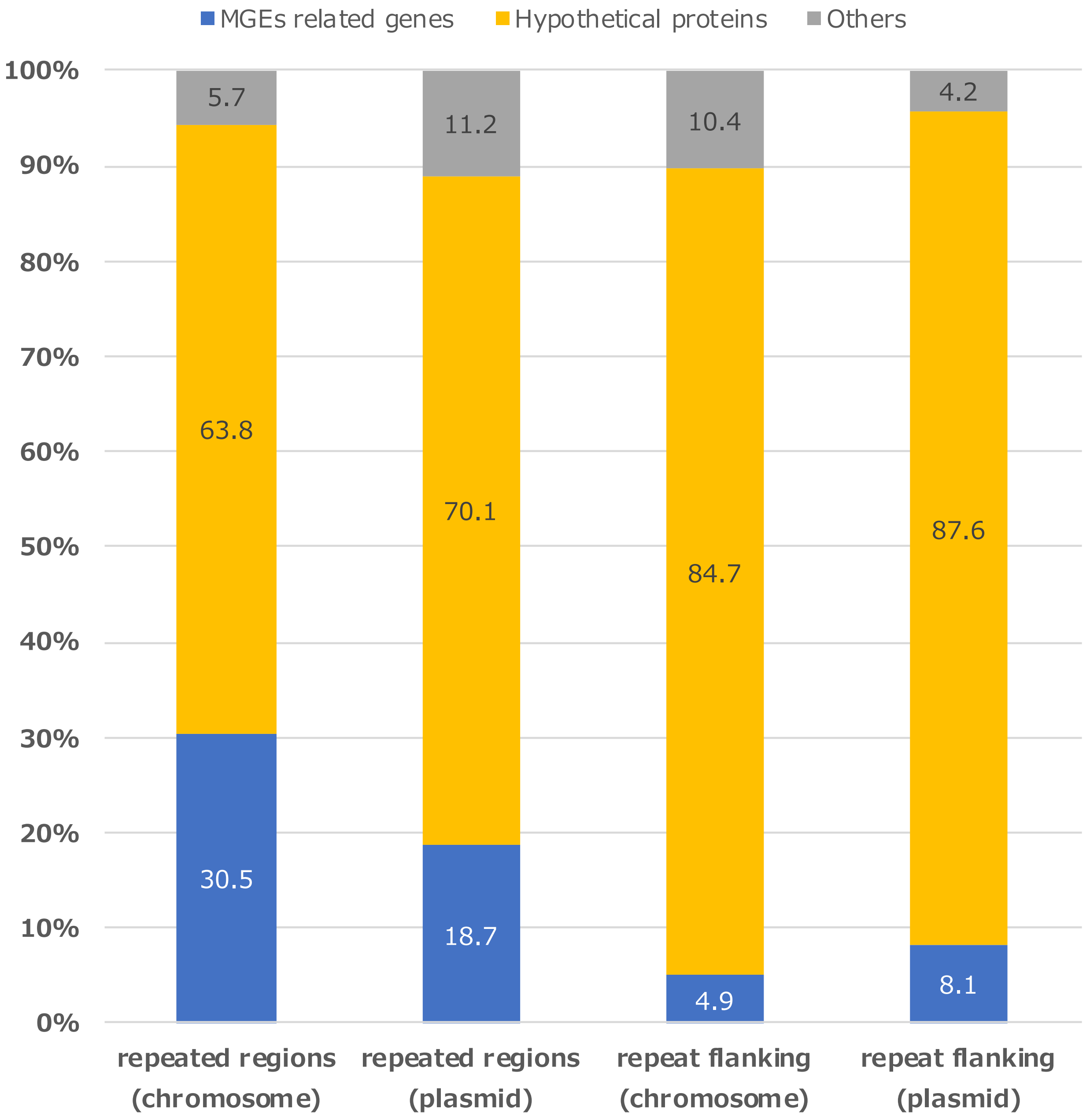

Supplementary Figure S2. Annotation results for coding sequences in repeated and repeat-flanking regions. The bars in the figure show the percentage of annotations of coding sequences in the repeat region and its neighboring regions detected in chromosomes and plasmids. The color of the bars corresponds to the annotation results. Blue indicates mobile genetic elements (MGEs) related genes, orange indicates hypothetical proteins, and pink indicates others.

Table S1. Results of phylogenetic classification of 16S rRNA genes.

Strain	sequence_number	sequence_identifier	sequence_score	bp_score	cutoff_head	cutoff_tail	identity	quality	startpos	stoppos	ecolipos	bps	gene_bps	lca_tax_slv
FNDCR1	1	16S_rRNA&a&acontig1_3491337_1.00x_true&a62734-64219&c+&d	0.999474	119	3	13	100	99	1003	43282	4	1485	1484	Bacteria;Proteobacteria;Alphaproteobacteria;Acetobacterales;Acetobacteraceae;Komagataeibacter;
FNDCR1	2	16S_rRNA&a&acontig1_3491337_1.00x_true&a2846923-2848408&c+&d	0.999474	119	3	13	100	99	1003	43282	4	1485	1484	Bacteria;Proteobacteria;Alphaproteobacteria;Acetobacterales;Acetobacteraceae;Komagataeibacter;
FNDCR1	3	16S_rRNA&a&acontig1_3491337_1.00x_true&a1214450-1215935&c-&d	0.999474	119	3	13	100	99	1003	43282	4	1485	1484	Bacteria;Proteobacteria;Alphaproteobacteria;Acetobacterales;Acetobacteraceae;Komagataeibacter;
FNDCR1	4	16S_rRNA&a&acontig1_3491337_1.00x_true&a1635916-1637401&c-&d	0.999474	119	3	13	100	99	1003	43282	4	1485	1484	Bacteria;Proteobacteria;Alphaproteobacteria;Acetobacterales;Acetobacteraceae;Komagataeibacter;
FNDCR1	5	16S_rRNA&a&acontig1_3491337_1.00x_true&a2257658-2259143&c-&d	0.999474	119	3	13	100	99	1003	43282	4	1485	1484	Bacteria;Proteobacteria;Alphaproteobacteria;Acetobacterales;Acetobacteraceae;Komagataeibacter;
FNDCF1	1	16S_rRNA&a&acontig1_3003528_1.00x_true&a61595-63080&c+&d	0.999472	119	3	13	99.1156	99	1003	43282	4	1485	1484	Bacteria;Proteobacteria;Alphaproteobacteria;Acetobacterales;Acetobacteraceae;Komagataeibacter;
FNDCF1	2	16S_rRNA&a&acontig1_3003528_1.00x_true&a2369282-2370767&c+&d	0.999472	119	3	13	99.1156	99	1003	43282	4	1485	1484	Bacteria;Proteobacteria;Alphaproteobacteria;Acetobacterales;Acetobacteraceae;Komagataeibacter;
FNDCF1	3	16S_rRNA&a&acontig1_3003528_1.00x_true&a2862389-2863874&c+&d	0.999472	119	3	13	99.1156	99	1003	43282	4	1485	1484	Bacteria;Proteobacteria;Alphaproteobacteria;Acetobacterales;Acetobacteraceae;Komagataeibacter;
FNDCF1	4	16S_rRNA&a&acontig1_3003528_1.00x_true&a1483754-1485239&c-&d	0.999472	119	3	13	99.1156	99	1003	43282	4	1485	1484	Bacteria;Proteobacteria;Alphaproteobacteria;Acetobacterales;Acetobacteraceae;Komagataeibacter;
FNDCF1	5	16S_rRNA&a&acontig1_3003528_1.00x_true&a1861151-1862636&c-&d	0.999472	119	3	13	99.1156	99	1003	43282	4	1485	1484	Bacteria;Proteobacteria;Alphaproteobacteria;Acetobacterales;Acetobacteraceae;Komagataeibacter;
FNDCR2	1	16S_rRNA&a&acontig1_3528188_1.00x_true&a63087-64572&c+&d	0.999473	119	3	13	99.5854	99	1003	43282	4	1485	1484	Bacteria;Proteobacteria;Alphaproteobacteria;Acetobacterales;Acetobacteraceae;Komagataeibacter;
FNDCR2	2	16S_rRNA&a&acontig1_3528188_1.00x_true&a2935104-2936589&c+&d	0.999473	119	3	13	99.5854	99	1003	43282	4	1485	1484	Bacteria;Proteobacteria;Alphaproteobacteria;Acetobacterales;Acetobacteraceae;Komagataeibacter;
FNDCR2	3	16S_rRNA&a&acontig1_3528188_1.00x_true&a3386204-3387689&c+&d	0.999473	119	3	13	99.5854	99	1003	43282	4	1485	1484	Bacteria;Proteobacteria;Alphaproteobacteria;Acetobacterales;Acetobacteraceae;Komagataeibacter;
FNDCR2	4	16S_rRNA&a&acontig1_3528188_1.00x_true&a1951256-1952741&c-&d	0.999473	119	3	13	99.5854	99	1003	43282	4	1485	1484	Bacteria;Proteobacteria;Alphaproteobacteria;Acetobacterales;Acetobacteraceae;Komagataeibacter;
FNDCR2	5	16S_rRNA&a&acontig1_3528188_1.00x_true&a2399186-2400671&c-&d	0.999473	119	3	13	99.5854	99	1003	43282	4	1485	1484	Bacteria;Proteobacteria;Alphaproteobacteria;Acetobacterales;Acetobacteraceae;Komagataeibacter;

Table S2. Observed genes in the repeated and repeat flanking regions in *Komagataeibacter* genomes.

	Chromosomes		Plasmids	
	within repeated region	repeat flanking region	within repeated region	repeat flanking region
1,4-alpha-glucan branching enzyme GlgB	3	0	0	0
2-(hydroxymethyl)glutarate dehydrogenase	3	0	0	0
2-ketogluconate reductase	0	2	0	0
2-methoxy-6-polyprenyl-1,4-benzoquinol methylase, mitochondrial	3	0	0	0
3'(2'),5'-bisphosphate nucleotidase CysQ	3	0	0	0
3-oxoacyl-[acyl-carrier-protein] reductase FabG	0	0	2	0
3-phenylpropionate-dihydrodiol/cinnamic acid-dihydrodiol dehydrogenase	1	0	0	0
34 kDa membrane antigen	3	12	0	0
4,4'-diaponeurosporen-aldehyde dehydrogenase	0	0	2	0
4-alpha-glucanotransferase	3	0	0	0
4-hydroxy-3-methylbut-2-en-1-yl diphosphate synthase (ferredoxin)	1	0	0	0
4-hydroxy-3-methylbut-2-en-1-yl diphosphate synthase (flavodoxin)	5	0	0	0
5-methyltetrahydropteroyltriglutamate--homocysteine methyltransferase	0	0	2	0
6-phosphogluconate dehydrogenase, NAD(+)-dependent, decarboxylating	3	0	0	0
6-phosphogluconolactonase	5	0	0	0
8-amino-7-oxononanoate synthase	3	0	0	0
ABC transporter ATP-binding protein uup	2	0	0	0
ATP-dependent RNA helicase CshA	1	0	0	0
ATP-dependent RNA helicase SrmB	0	0	3	0
ATP-dependent RecD-like DNA helicase	0	3	6	0
Acetyl-coenzyme A synthetase	3	0	0	0
Adaptive-response sensory-kinase SasA	0	0	1	0
Adenine deaminase	3	0	0	0
Adenosylmethionine-8-amino-7-oxononanoate aminotransferase	3	0	0	0
Adenylosuccinate synthetase	0	2	0	0
Aerobic C4-dicarboxylate transport protein	0	0	2	0
Aerobic cobaltochelatase subunit CobS	3	0	0	0
Aerobic cobaltochelatase subunit CobT	3	0	0	0
Aldehyde dehydrogenase	0	1	0	0
Aliphatic amidase expression-regulating protein	3	0	0	0
Alpha-hemolysin translocation ATP-binding protein HlyB	2	0	0	0
Amidophosphoribosyltransferase	6	0	0	0
Aminopyrimidine aminohydrolase	0	1	0	0
Antitoxin DinJ	0	0	5	0
Antitoxin FitA	0	0	3	0
Antitoxin VapB	0	0	1	0
Arabinose 5-phosphate isomerase KdsD	3	0	0	0
Arsenate reductase	0	0	1	0
Arsenical pump membrane protein	0	0	1	0
Aspartate kinase Ask_LysC	3	0	0	0
Aspartokinase	3	0	0	0
Bacterioferritin	2	4	0	0
Beta-barrel assembly-enhancing protease	3	0	0	0
Bifunctional transcriptional activator/DNA repair enzyme AdaA	0	0	0	1
Bifunctional uridylyltransferase/uridylyl-removing enzyme	3	0	0	0
Biopolymer transport protein ExbD	6	0	0	0
Branched-chain-amino-acid aminotransferase	3	0	0	0
C4-dicarboxylic acid transporter DauA	0	0	3	0
CDP-diacylglycerol pyrophosphatase	3	0	0	0
Caffeate CoA-transferase	0	4	0	0
Cardiolipin synthase A	0	2	0	0
Cellulose synthase 1	9	0	0	0
Cellulose synthase operon protein C	9	0	0	0
Cellulose synthase operon protein D	0	4	0	0
Chaperone protein ClpB	0	86	0	0
Chaperone protein DnaJ	3	0	0	0
Chaperone protein DnaK	3	0	0	0
Chromosome-partitioning ATPase Soj	0	0	3	0
Cobalt-precorrin-5B C(1)-methyltransferase	3	0	0	0
Cobalt-zinc-cadmium resistance protein CzcA	0	0	10	0
Colicin V production protein	3	0	0	0
Competence protein ComM	1	0	0	0
Coniferyl aldehyde dehydrogenase	0	0	1	0
Copper-exporting P-type ATPase	0	0	3	0
Copper-transporting P-type ATPase	0	0	3	0
Cyclic-di-GMP-binding biofilm dispersal mediator protein	0	0	2	0
Cys-tRNA(Pro)/Cys-tRNA(Cys) deacylase YbaK	3	0	0	0
Cysteine--tRNA ligase	0	6	0	0
Cytochrome bd-II ubiquinol oxidase subunit 2	0	5	0	0
Cytochrome c oxidase subunit 1	3	0	0	0
Cytochrome c oxidase subunit 1 , bacteroid	0	0	3	0
Cytosol aminopeptidase	3	0	0	0
D-inositol-3-phosphate glycosyltransferase	6	0	0	0
DNA adenine methyltransferase YhdJ	3	0	0	0
DNA helicase II	0	0	3	0
DNA mismatch repair protein MutS	3	0	0	0
DNA primase TraC	0	0	6	0

Table S2 (1/5)

DNA repair protein RadA	0	5	0	0
DNA-binding protein HU-beta	0	4	0	0
DNA-binding transcriptional regulator BolA	2	0	0	0
DNA-directed RNA polymerase subunit omega	3	0	0	0
DNA-invertase hin	0	0	71	0
Dihydrolipoyl dehydrogenase 3	0	1	0	0
Dimethyl-sulfide monooxygenase	0	3	0	0
Dipeptide transport system permease protein DppB	1	0	0	0
Disulfide-bond oxidoreductase YfcG	0	0	0	4
Divalent metal cation transporter MntH	3	0	0	0
Elongation factor 4	1	1	0	0
Endoribonuclease toxin MazF	1	0	0	0
Energy-dependent translational throttle protein EttA	3	0	0	0
Error-prone DNA polymerase	0	25	3	2
Exodeoxyribonuclease 7 large subunit	3	0	0	0
FMN reductase (NADPH)	3	0	0	0
Farnesyl diphosphate synthase	0	8	0	0
Fe(2+) transporter FeoB	3	0	0	0
Ferric uptake regulation protein	1	0	0	0
Flavin-dependent monooxygenase	1	0	0	0
Flavin-dependent thymidylate synthase	0	0	3	0
Flavohemoprotein	0	0	3	0
Fructose-bisphosphate aldolase class 1	3	0	0	0
FtsZ-localized protein A	0	0	2	0
Galactose-proton symporter	3	0	0	0
Glucose-6-phosphate 1-dehydrogenase 2	3	4	0	0
Glutamate synthase [NADPH] large chain	3	0	0	0
Glutamate synthase [NADPH] small chain	3	0	0	0
Glutamine synthetase	3	0	0	0
Glutathione peroxidase BsaA	3	0	0	0
Glutathione synthetase	3	0	0	0
Glutathione transport system permease protein GsiD	1	0	0	0
Glycerol dehydrogenase small subunit	0	3	0	0
Glycerol-3-phosphate dehydrogenase [NAD(P)+]	3	0	0	0
Glycogen operon protein GlgX	3	0	0	0
Glycogen phosphorylase	3	0	0	0
Glycogen synthase 1	3	0	0	0
Guanine/hypoxanthine permease GhxP	0	3	0	0
Guanine/hypoxanthine permease GhxQ	0	2	0	0
H(+)/Cl(-) exchange transporter ClcA	0	0	15	0
HTH-type transcriptional regulator DmlR	0	0	1	0
HTH-type transcriptional regulator HdfR	0	0	2	0
HTH-type transcriptional regulator HmrR	0	0	6	0
HTH-type transcriptional regulator IscR	2	0	0	0
HTH-type transcriptional repressor NicR	0	0	3	0
HTH-type transcriptional repressor NsrR	1	0	0	1
Haloacetate dehalogenase H-1	0	0	3	0
Hemolysin secretion protein D, plasmid	6	0	0	0
High-affinity branched-chain amino acid transport ATP-binding protein LivF	3	0	0	0
High-affinity nickel transport protein	1	0	0	0
Histidine--tRNA ligase	3	0	0	0
Hydrogenobyrinate a,c-diamide synthase	3	0	0	0
Hydroxyacylglutathione hydrolase	0	1	0	0
Hydroxyacylglutathione hydrolase GloB	0	1	0	0
IS110 family transposase ISAzs4	1	0	2	0
IS110 family transposase ISCc4	70	0	32	0
IS110 family transposase ISHne5	1	2	0	2
IS110 family transposase ISMdi12	34	0	11	0
IS110 family transposase ISPye26	0	0	4	0
IS110 family transposase ISRel9	4	0	2	0
IS110 family transposase ISRsp4	1	0	0	0
IS110 family transposase ISSfr2	0	0	7	0
IS110 family transposase ISShsp1	1	0	0	0
IS1182 family transposase ISGdi13	680	13	20	0
IS1380 family transposase IS1380A	0	0	1	0
IS1380 family transposase ISMesp2	0	0	3	0
IS1380 family transposase ISNha3	1	0	19	0
IS200/IS605 family transposase ISAba30	0	0	1	0
IS21 family transposase ISGdi17	115	21	5	0
IS21 family transposase ISPkr1	2	0	11	0
IS21 family transposase ISRel3	667	0	4	0
IS256 family transposase ISPye43	2	5	11	0
IS256 family transposase ISRm3	23	0	0	0
IS256 family transposase ISSpwi2	1	6	0	0
IS3 family transposase ISAli4	10	0	0	0
IS3 family transposase ISAli5	1	0	0	0
IS3 family transposase ISGxy1	78	25	56	4
IS3 family transposase ISMamg1	3	0	0	0
IS3 family transposase ISMtsp5	0	6	0	0
IS3 family transposase ISOgr1	0	2	0	0

Table S2 (2/5)

IS3 family transposase ISPam3	0	0	2	0
IS3 family transposase ISPfe1	1	9	0	0
IS3 family transposase ISPye52	0	1	0	0
IS3 family transposase ISRpa2	20	5	12	1
IS3 family transposase ISShsp3	26	0	0	0
IS3 family transposase ISXau3	3	0	0	0
IS4 family transposase ISPye60	1	1	0	0
IS481 family transposase ISBxe4	0	0	1	0
IS5 family transposase IS1031A	1	0	0	0
IS5 family transposase IS1032	0	32	0	8
IS5 family transposase IS1248A	0	0	1	0
IS5 family transposase ISAli12B	4	7	0	0
IS5 family transposase ISAli9	0	0	0	1
IS5 family transposase ISAzs9	4	18	0	0
IS5 family transposase ISGdi1	1	0	0	0
IS5 family transposase ISGdi3	3	12	0	4
IS5 family transposase ISMex35	53	0	15	3
IS5 family transposase ISMex42	0	3	0	0
IS5 family transposase ISPak1	0	47	2	0
IS5 family transposase ISPha1	0	2	0	0
IS5 family transposase ISPko1	63	0	10	0
IS5 family transposase ISPye24	5	5	2	0
IS5 family transposase ISPye39	1	0	0	0
IS5 family transposase ISPze1	0	1	0	0
IS5 family transposase ISTasp1	0	1	0	0
IS630 family transposase ISBj5	1	0	6	0
IS630 family transposase ISGdi4	388	2	32	6
IS630 family transposase ISGdi5	1	21	2	8
IS630 family transposase ISMex41	4	3	0	0
IS630 family transposase ISRm10-1	0	14	7	2
IS66 family transposase ISAli10	61	0	39	0
IS66 family transposase ISAzs20	2	8	3	7
IS66 family transposase ISAzs21	186	0	3	1
IS66 family transposase ISCARN48	190	0	38	0
IS701 family transposase IS1452	16	0	2	0
IS701 family transposase ISGdi12	3	0	4	1
IS701 family transposase ISNha1	3	0	1	0
IS91 family transposase ISAzo26	16	0	1	0
IS91 family transposase ISMno23	7	0	0	0
IS91 family transposase ISMno24	22	0	1	0
ISL3 family transposase ISAzba9	16	0	6	0
ISL3 family transposase ISAzs13	0	0	1	0
Inorganic pyrophosphatase	3	0	0	0
Insertion element IS6110 uncharacterized 12.0 kDa protein	0	0	0	3
Iron(3+)-hydroxamate import ATP-binding protein FhuC	3	0	0	0
Iron-sulfur cluster carrier protein	0	0	4	0
Isochorismatase family protein YecD	0	0	3	0
Isopentenyl-diphosphate delta-isomerase	3	0	0	0
Isoprimeverose transporter	0	6	0	0
L-glyceraldehyde 3-phosphate reductase	3	0	0	0
Levodione reductase	3	0	0	0
Lignostilbene-alpha,beta-dioxygenase isozyme I	0	3	0	0
Low affinity potassium transport system protein kup	3	0	0	0
Magnesium transport protein CorA	0	6	0	0
Malate:quinone oxidoreductase	3	0	0	0
Maleamate amidohydrolase	0	15	0	0
Malto-oligosyltrehalose trehalohydrolase	3	0	0	0
Membrane-bound lytic murein transglycosylase C	0	0	3	0
Metalloprotease PmbA	3	0	0	0
Methionine synthase	3	0	0	0
Methionine--tRNA ligase	0	1	0	0
Methylamine utilization protein MauG	3	0	0	0
Modification methylase RsrI	0	0	3	0
Multicopper oxidase MmcO	0	0	0	4
Multidrug resistance protein MdtA	6	0	0	0
Multidrug resistance protein MdtC	0	4	0	0
N-acetylmuramoyl-L-alanine amidase AmiA	0	1	0	0
N-ethylmaleimide reductase	2	0	0	0
NADH dehydrogenase	2	0	0	0
NADH:quinone reductase	3	0	0	0
NADPH-dependent FMN reductase ArsH	0	0	1	0
Na(+)/H(+) antiporter NhaP	0	0	6	0
Nickel and cobalt resistance protein CnrA	0	0	0	1
Non-heme chloroperoxidase	0	3	0	0
Octanoyltransferase	3	0	0	0
Oligopeptide transport ATP-binding protein OppD	1	0	0	0
Outer membrane protein	3	2	0	0
Oxygen sensor protein DosP	2	56	0	0
Peptide chain release factor 1	3	0	0	0
Phosphate acetyltransferase	3	0	0	0

Table S2 (3/5)

Phosphate-specific transport system accessory protein PhoU	0	2	0	0
Phosphoglucosamine mutase	3	0	0	0
Phosphomannomutase/phosphoglucomutase	2	0	0	0
Phosphomethylpyrimidine synthase	3	0	0	0
Phosphoribosylamine--glycine ligase	3	0	0	0
Phosphoribosylformylglycinamidine synthase subunit PurL	3	0	0	0
Polyphosphate:ADP phosphotransferase	2	0	0	0
Porin B	1	0	1	0
Porphobilinogen deaminase	3	0	0	0
Precorrin-2 C(20)-methyltransferase	3	0	0	0
Precorrin-3B C(17)-methyltransferase	3	0	0	0
Precorrin-4 C(11)-methyltransferase	3	0	0	0
Precorrin-6A reductase	3	0	0	0
Precorrin-6Y C(5,15)-methyltransferase [decarboxylating]	3	0	0	0
Precorrin-8X methylmutase	3	0	0	0
Protein ImuA	0	0	7	0
Protein ImuB	0	0	2	0
Protein adenylyltransferase SoFic	0	0	0	1
Protein tas	0	1	0	0
Protein-export protein SecB	3	0	0	0
Protoheme IX farnesyltransferase	3	0	0	0
Putative aminoacrylate hydrolase RutD	0	0	2	0
Putative monooxygenase MoxC	0	1	0	0
Putative pyruvate, phosphate dikinase regulatory protein	0	64	0	0
Putative signal peptide peptidase SppA	3	0	0	0
Pyridoxal phosphate homeostasis protein	3	0	0	0
Pyridoxine 4-dehydrogenase	0	0	2	0
Pyrimidine-specific ribonucleoside hydrolase RihA	3	0	0	0
Quinone oxidoreductase 1	0	7	0	0
Quinoprotein glucose dehydrogenase	1	0	0	0
RNA polymerase sigma factor RpoH	1	0	0	0
RNA-binding protein Hfq	0	2	0	0
Regulatory protein SdiA	0	12	0	0
Release factor glutamine methyltransferase	3	0	0	0
Replicative DNA helicase	4	0	0	0
Riboflavin transporter RibZ	3	0	0	0
Ribonuclease D	3	0	0	0
Ribonuclease VapC11	0	0	0	1
Ribose import ATP-binding protein RbsA	3	0	0	0
Ribose operon repressor	0	2	0	0
Ribosomal RNA large subunit methyltransferase J	0	6	0	0
Ribosomal large subunit pseudouridine synthase D	2	0	0	0
S-(hydroxymethyl)glutathione dehydrogenase	4	0	0	0
S-adenosylmethionine synthase	0	2	0	0
S-formylglutathione hydrolase FrmB	3	0	0	0
Sec-independent protein translocase protein TatB	1	0	0	0
Sec-independent protein translocase protein TatC	1	0	0	0
Segregation and condensation protein A	3	0	1	0
Segregation and condensation protein B	1	0	0	0
Sensor histidine kinase CusS	0	7	0	0
Sensor histidine kinase RcsC	6	0	0	0
Serine hydroxymethyltransferase 2	2	0	0	0
Serine--tRNA ligase	1	0	0	0
Silver exporting P-type ATPase	0	0	3	0
Single-stranded DNA-binding protein	0	0	6	0
Sporulation initiation inhibitor protein Soj	0	0	3	0
Succinate-semialdehyde dehydrogenase [NADP(+)] 1	2	0	0	0
Sugar phosphatase YidA	8	2	0	0
Sulfate transport system permease protein CysW	2	0	0	0
Sulfate-binding protein	2	0	0	0
Sulfoacetaldehyde reductase 2	0	0	0	1
Thermostable carboxypeptidase 1	3	0	0	0
Thioredoxin reductase	0	30	0	0
Thiosulfate dehydrogenase	0	0	3	0
Threonine synthase	3	0	0	0
Threonylcarbamoyl-AMP synthase	3	0	0	0
Tn3 family transposase	0	0	54	0
Tn3 family transposase ISAli20	0	0	14	0
Tn3 family transposase ISAzs17	0	0	9	0
Tn3 family transposase ISMpo10	0	0	1	0
Tol-Pal system protein TolQ	3	0	0	0
Toxin Doc	0	0	3	0
Toxin FitB	0	0	3	0
Transaldolase	3	0	0	0
Transcription elongation factor GreB	3	0	0	0
Transcriptional activator protein CopR	0	0	1	0
Transcriptional regulator SlyA	1	0	0	0
Transcriptional regulatory protein BaeR	0	4	0	0
Transcriptional regulatory protein QseB	0	0	1	0
Transcriptional regulatory protein ros	0	2	0	0

Table S2 (4/5)

Transcriptional repressor FrmR	3	0	0	0
Transketolase	3	0	0	0
Transposon Tn3 resolvase	0	0	2	0
Tryptophan decarboxylase	3	0	0	0
Tyrosine recombinase XerC	6	3	12	0
UDP-glucose 4-epimerase	2	0	0	0
UTP--glucose-1-phosphate uridylyltransferase	3	0	0	0
Ubiquinone biosynthesis O-methyltransferase	3	0	0	0
Ubiquinone hydroxylase UbiM	0	95	0	0
Urease accessory protein UreD	3	0	0	0
Urease accessory protein UreE	3	0	0	0
Urease accessory protein UreF	3	0	0	0
Urease accessory protein UreG	3	0	0	0
Urease subunit alpha 1	3	0	0	0
Urease subunit beta 1	3	0	0	0
Urease subunit gamma	3	0	0	0
Uric acid transporter UacT	3	0	0	0
UvrABC system protein B	3	0	0	0
Validamycin A dioxygenase	0	0	3	0
Vitamin B12 import ATP-binding protein BtuD	5	0	0	0
Vitamin B12 import system permease protein BtuC	3	0	0	0
Vitamin B12 transporter BtuB	4	0	0	0
Zinc-type alcohol dehydrogenase-like protein	0	0	6	0
hypothetical protein	5857	4659	1720	517
mRNA interferase toxin RelE	0	7	0	0
mRNA interferase toxin YafQ	0	0	5	0
phosphoketolase	0	12	0	0
protein kinase UbiB	3	0	0	0
putative ABC transporter ATP-binding protein	2	0	0	0
putative MFS-type transporter YfcJ	0	4	0	0
putative acrylyl-CoA reductase AcuI	3	0	0	0
putative amino acid permease YhdG	1	0	0	0
putative endoglucanase	2	0	0	0
putative glycosyltransferase YkoT	2	0	0	0
putative lipid II flippase MurJ	3	0	0	0
putative oxidoreductase	0	0	0	4
putative oxidoreductase CzcO	2	0	0	0
putative oxidoreductase YciK	3	0	0	0
putative oxidoreductase YjmC	0	0	2	0
putative oxidoreductase/MSMEI_2347	3	0	0	0
putative protein	3	0	0	0
putative protein YphB	3	0	0	0
putative soluble pyridine nucleotide transhydrogenase	0	0	3	0
putative transporter YycB	0	0	2	0
putative zinc protease	3	0	0	0
scyllo-inositol 2-dehydrogenase (NAD(+))	2	0	0	0
tRNA 5-hydroxyuridine methyltransferase	6	9	0	0
tRNA N6-adenosine threonylcarbamoyltransferase	3	0	0	0
tRNA(fMet)-specific endonuclease VapC	0	8	4	2

Table S2 (5/5)

Table S3. Annotation results in the cellulose biosynthesis operon.

FNDCR1 strain

Start	Stop	Length	Strand	Annotation	Gene name	Comments
2082503	2087920	5418	-	ID=fig|6666666.451602.peg.2163;Name=hypothetical protein (similar to bcsC)	*bcsCII*	
2087955	2089106	1152	-	ID=fig|6666666.451602.peg.2164;Name=O-antigen acetylase (similar to bcsY)	*bcsY*	
2089186	2089869	684	-	ID=fig|6666666.451602.peg.2165;Name=hypothetical protein (similar to bcsX)	*bcsX*	
2089939	2090055	117	+	ID=fig|6666666.451602.peg.2166;Name=hypothetical protein	*orf2166*	
2090052	2094839	4788	-	ID=fig|6666666.451602.peg.2167;Name=Cellulose synthase catalytic subunit [UDP-forming] (EC 2.4.1.12);Ontology_term=KEGG_ENZYME:2.4.1.12	*bcsABII*	
3021847	3024027	2181	+	ID=fig|6666666.451602.peg.3166;Name=hypothetical protein (similar to beta-glucosidase B)	*bglX_1*	
3176919	3177917	999	+	ID=fig|6666666.451602.peg.3317;Name=beta-1%2C4-glucanase (cellulase) (EC 3.2.1.4);Ontology_term=KEGG_ENZYME:3.2.1.4	*bcsZ_1*	
3183315	3184313	999	+	ID=fig|6666666.451602.peg.3324;Name=beta-1%2C4-glucanase (cellulase) (EC 3.2.1.4);Ontology_term=KEGG_ENZYME:3.2.1.4	*bcsZ_2*	100% identical to *bcsZ_1* in nucleotide sequence
3184310	3185428	1119	+	ID=fig|6666666.451602.peg.3325;Name=hypothetical protein (similar to cellulose-complementing protein A)	*bcsH*	
3185693	3190297	4605	+	ID=fig|6666666.451602.peg.3326;Name=Cellulose synthase catalytic subunit [UDP-forming] (EC 2.4.1.12);Ontology_term=KEGG_ENZYME:2.4.1.12	*bcsABI*	
3190294	3194196	3903	+	ID=fig|6666666.451602.peg.3327;Name=hypothetical protein (similar to bcsC)	*bcsCI*	
3194196	3194666	471	+	ID=fig|6666666.451602.peg.3328;Name=Cellulose synthase operon protein D	*bcsDI*	
3194702	3194872	171	-	ID=fig|6666666.451602.peg.3329;Name=hypothetical protein	*orf3329*	
3194995	3195354	360	+	ID=fig|6666666.451602.peg.3330;Name=hypothetical protein (similar to IS66 family transposase)	*IS_1*	
3195351	3195698	348	+	ID=fig|6666666.451602.peg.3331;Name=Mobile element protein (similar to IS66 family insertion sequence element accessory protein)	*IS_2*	
3195813	3197372	1560	+	ID=fig|6666666.451602.peg.3332;Name=Mobile element protein (similar to IS66 family transposase)	*IS_3*	
3197543	3199759	2217	+	ID=fig|6666666.451602.peg.3333;Name=beta-glucosidase (EC 3.2.1.21);Ontology_term=KEGG_ENZYME:3.2.1.21	*bglX_2*	
3199777	3200490	714	+	ID=fig|6666666.451602.peg.3334;Name=Tiorf138 protein	*orf3334*	
3200690	3201184	495	+	ID=fig|6666666.451602.peg.3335;Name=FIG018171: hypothetical protein of Cupin superfamily	*orf3335*	
3201184	3201834	651	+	ID=fig|6666666.451602.peg.3336;Name=hydrolase%2C NUDIX family	*orf3336*	
3201968	3203839	1872	+	ID=fig|6666666.451602.peg.3337;Name=Chaperone protein HtpG	*htpG*	

FNDCF1 strain

Start	Stop	Length	Strand	Annotation	Gene name	Comments
215017	217797	2781	+	ID=fig|6666666.451604.peg.215;Name=beta-glucosidase (EC 3.2.1.21);Ontology_term=KEGG_ENZYME:3.2.1.21	*bglX_1*	
2443500	2445536	2037	+	ID=fig|6666666.451604.peg.2448;Name=hypothetical protein (similar to beta-glucosidase B)	*bglX_2*	
2588758	2589429	672	+	ID=fig|6666666.451604.peg.2583;Name=hypothetical protein (similar to bcsX)	*bcsX_1*	
2589508	2590668	1161	+	ID=fig|6666666.451604.peg.2584;Name=O-antigen acetylase (similar to bcsY)	*bcsY*	
2590685	2594272	3588	+	ID=fig|6666666.451604.peg.2585;Name=hypothetical protein (similar to bcsC, partial gene)	*bcsCII/N*	Truncated form N-terminus
2594424	2594555	132	+	ID=fig|6666666.451604.peg.2586;Name=hypothetical protein (similar to bcsC, partial gene)	*bcsCII/C*	Rest of the above polypeptide
2594561	2596279	1719	+	ID=fig|6666666.451604.peg.2587;Name=hypothetical protein (similar to bcsX)	*bcsX_2*	
2596403	2597500	1098	+	ID=fig|6666666.451604.peg.2588;Name=beta-1%2C4-glucanase (cellulase) (EC 3.2.1.4);Ontology_term=KEGG_ENZYME:3.2.1.4	*bcsZ*	
2597497	2598651	1155	+	ID=fig|6666666.451604.peg.2589;Name=hypothetical protein (similar to cellulose-complementing protein A)	*bcsH*	
2598916	2603493	4578	+	ID=fig|6666666.451604.peg.2590;Name=Cellulose synthase catalytic subunit [UDP-forming] (EC 2.4.1.12);Ontology_term=KEGG_ENZYME:2.4.1.12	*bcsABI*	
2603700	2604170	471	+	ID=fig|6666666.451604.peg.2591;Name=Cellulose synthase operon protein D	*bcsDI*	
2604185	2604343	159	+	ID=fig|6666666.451604.peg.2592;Name=hypothetical protein	*orf2592*	
2604422	2605090	669	+	ID=fig|6666666.451604.peg.2593;Name=Tiorf138 protein	*orf2593*	
2605177	2605635	459	+	ID=fig|6666666.451604.peg.2594;Name=FIG018171: hypothetical protein of Cupin superfamily	*orf2594*	
2605632	2606270	639	+	ID=fig|6666666.451604.peg.2595;Name=NUDIX hydrolase	*orf2595*	
2606368	2608251	1884	+	ID=fig|6666666.451604.peg.2596;Name=Chaperone protein HtpG	*htpG*	

FNDCR2 strain

Start	Stop	Length	Strand	Annotation	Gene name	Comments
1241400	1245593	4194	-	ID=fig|6666666.452812.peg.1321;Name=Cellulose synthase catalytic subunit [UDP-forming] (EC 2.4.1.12);Ontology_term=KEGG_ENZYME:2.4.1.12	*bcsABII*	
3118049	3119128	1080	+	ID=fig|6666666.452812.peg.3223;Name=beta-1%2C4-glucanase (cellulase) (EC 3.2.1.4);Ontology_term=KEGG_ENZYME:3.2.1.4	*bcsZ*	
3119125	3120135	1011	+	ID=fig|6666666.452812.peg.3224;Name=hypothetical protein (similar to cellulose-complementing protein A)	*bcsH*	
3120158	3120271	114	+	ID=fig|6666666.452812.peg.3225;Name=hypothetical protein	*orf3225*	
3120318	3122555	2238	+	ID=fig|6666666.452812.peg.3226;Name=Cellulose synthase catalytic subunit [UDP-forming] (EC 2.4.1.12);Ontology_term=KEGG_ENZYME:2.4.1.12	*bcsAI*	
3122542	3124968	2427	+	ID=fig|6666666.452812.peg.3227;Name=hypothetical protein (similar to bcsB)	*bcsBI*	
3124971	3128948	3978	+	ID=fig|6666666.452812.peg.3228;Name=hypothetical protein (similar to bcsC)	*bcsCI*	
3128948	3129418	471	+	ID=fig|6666666.452812.peg.3229;Name=Cellulose synthase operon protein D	*bcsDI*	
3129466	3129594	129	+	ID=fig|6666666.452812.peg.3869;Name=hypothetical protein	*orf3869*	
3129632	3131830	2199	+	ID=fig|6666666.452812.peg.3230;Name=beta-glucosidase (EC 3.2.1.21);Ontology_term=KEGG_ENZYME:3.2.1.21	*bglX*	
3131830	3132495	666	+	ID=fig|6666666.452812.peg.3231;Name=Tiorf138 protein	*orf3231*	
3132671	3133129	459	+	ID=fig|6666666.452812.peg.3232;Name=FIG018171: hypothetical protein of Cupin superfamily	*orf3232*	
3133126	3133764	639	+	ID=fig|6666666.452812.peg.3233;Name=hydrolase%2C NUDIX family	*orf3233*	
3133860	3135743	1884	+	ID=fig|6666666.452812.peg.3234;Name=Chaperone protein HtpG	*htpG*	
3398218	3400338	2121	+	ID=fig|6666666.452812.peg.3487;Name=Cellulose synthase catalytic subunit [UDP-forming] (EC 2.4.1.12);Ontology_term=KEGG_ENZYME:2.4.1.12	*bcsAII*	
3400400	3400909	510	+	ID=fig|6666666.452812.peg.3488;Name=Mobile element protein (similar to IS630 family transposase)	*IS_1*	
3401110	3401466	357	+	ID=fig|6666666.452812.peg.3489;Name=Mobile element protein (similar to IS630 family transposase)	*IS_2*	
3401510	3403954	2445	+	ID=fig|6666666.452812.peg.3490;Name=hypothetical protein (similar to bcsB)	*bcsBII*	
3404107	3404937	831	-	ID=fig|6666666.452812.peg.3491;Name=Transposase (similar to IS5 family transposase)	*IS_3*	
3405109	3405663	555	+	ID=fig|6666666.453030.peg.3483;Name=hypothetical protein (similar to bcsX)	*bcsX*	Shorter than other homologues; truncated?
3405706	3406866	1161	+	ID=fig|6666666.453030.peg.3484;Name=O-antigen acetylase (similar to bcsY)	*bcsY*	
3406874	3410815	3942	+	ID=fig|6666666.452812.peg.3494;Name=hypothetical protein (similar to bcsC)	*bcsCII*	
3427119	3428141	1023	-	ID=fig|6666666.452812.peg.3511;Name=Endoglucanase	*bglC*	

NBRC3288 strain

Start	Stop	Length	Strand	Annotation	Gene name	Comments
903811	906993	3183	-	ID=fig|6666666.459276.peg.950;Name=Cellulose synthase operon protein C	*bcsCII_1*	
906972	909902	2931	-	ID=fig|6666666.459276.peg.951;Name=Cellulose synthase catalytic subunit [UDP-forming] (EC 2.4.1.12);Ontology_term=KEGG_ENZYME:2.4.1.12	*bcsABII_1*	
991034	995605	4572	-	ID=fig|6666666.459276.peg.1035;Name=Cellulose synthase catalytic subunit [UDP-forming] (EC 2.4.1.12);Ontology_term=KEGG_ENZYME:2.4.1.12	*bcsABII_2*	
2768782	2769861	1080	+	ID=fig|6666666.459276.peg.2900;Name=beta-1%2C4-glucanase (cellulase) (EC 3.2.1.4);Ontology_term=KEGG_ENZYME:3.2.1.4	*bcsZ*	
2769858	2770850	993	+	ID=fig|6666666.459276.peg.2901;Name=hypothetical protein (similar to cellulose-complementing protein A)	*bcsH*	Shorter than other homologues; truncated?
2770876	2770995	120	+	ID=fig|6666666.459276.peg.2902;Name=hypothetical protein	*orf2902*	
2771036	2773273	2238	+	ID=fig|6666666.459276.peg.2903;Name=Cellulose synthase catalytic subunit [UDP-forming] (EC 2.4.1.12);Ontology_term=KEGG_ENZYME:2.4.1.12	*bcsAI*	
2773260	2774816	1557	+	ID=fig|6666666.459276.peg.2904;Name=hypothetical protein (similar to bcsB)	*bcsBI/N*	Truncated form N-terminus
2774806	2775651	846	+	ID=fig|6666666.459276.peg.2905;Name=hypothetical protein (similar to bcsB)	*bcsBI/C*	Rest of the above polypeptide
2775654	2779661	4008	+	ID=fig|6666666.459276.peg.2906;Name=hypothetical protein (similar to bcsC)	*bcsCI*	
2779661	2780131	471	+	ID=fig|6666666.459276.peg.2907;Name=hypothetical protein (similar to bcsD)	*bcsDI*	
2780193	2780327	135	+	ID=fig|6666666.459276.peg.2908;Name=hypothetical protein	*orf2908*	
2780359	2782560	2202	+	ID=fig|6666666.459276.peg.2909;Name=beta-glucosidase (EC 3.2.1.21);Ontology_term=KEGG_ENZYME:3.2.1.21	*bglX*	
2782569	2783207	639	+	ID=fig|6666666.459276.peg.2910;Name=Tiorf138 protein	*orf2910*	
2783526	2783984	459	+	ID=fig|6666666.459276.peg.2911;Name=FIG018171: hypothetical protein of Cupin superfamily	*orf2911*	
2783981	2784619	639	+	ID=fig|6666666.459276.peg.2912;Name=hydrolase%2C NUDIX family	*orf2912*	
2784880	2786763	1884	+	ID=fig|6666666.459276.peg.2913;Name=Chaperone protein HtpG	*htpG*	
3057454	3062019	4566	+	ID=fig|6666666.459276.peg.3174;Name=Cellulose synthase catalytic subunit [UDP-forming] (EC 2.4.1.12);Ontology_term=KEGG_ENZYME:2.4.1.12	*bcsABII_3*	
3062128	3062796	669	+	ID=fig|6666666.459276.peg.3175;Name=hypothetical protein (similar to bcsX)	*bcsX_1*	
3063249	3064040	792	+	ID=fig|6666666.459276.peg.3176;Name=hypothetical protein (similar to bcsY)	*bcsY*	Shorter than other homologues; truncated?
3064048	3064410	363	+	ID=fig|6666666.459276.peg.3177;Name=hypothetical protein (similar to bcsC)	*bcsCII_2/N*	Truncated form N-terminus
3064487	3064846	360	+	ID=fig|6666666.459276.peg.3178;Name=hypothetical protein (similar to IS66 family transposase)	*IS_1*	
3064843	3065190	348	+	ID=fig|6666666.459276.peg.3179;Name=Mobile element protein (similar to IS66 family insertion sequence element accessory protein)	*IS_2*	
3065305	3066864	1560	+	ID=fig|6666666.459276.peg.3180;Name=Mobile element protein (similar to IS66 family transposase)	*IS_3*	
3066819	3070520	3702	+	ID=fig|6666666.459276.peg.3181;Name=hypothetical protein (similar to bcsC)	*bcsCII_2/C*	Rest of the above polypeptide
3070578	3072254	1677	+	ID=fig|6666666.459276.peg.3182;Name=hypothetical protein (similar to bcsX)	*bcsX_2*	
3088388	3089410	1023	-	ID=fig|6666666.459276.peg.3200;Name=Endoglucanase	*bglC*	

ATCC23769 strain

Start	Stop	Length	Strand	Annotation	Gene name	Comments
907546	908646	1101	+	ID=fig|6666666.459278.peg.983;Name=beta-1%2C4-glucanase (cellulase) (EC 3.2.1.4);Ontology_term=KEGG_ENZYME:3.2.1.4	*bcsZ*	
908643	909704	1062	+	ID=fig|6666666.459278.peg.984;Name=hypothetical protein (similar to cellulose-complementing protein A)	*bcsH*	
909735	909893	159	+	ID=fig|6666666.459278.peg.985;Name=hypothetical protein (SImilar to bcsAB)	*bcsABI/N*	Truncated form N-terminus?
909980	914581	4602	+	ID=fig|6666666.459278.peg.986;Name=Cellulose synthase catalytic subunit [UDP-forming] (EC 2.4.1.12);Ontology_term=KEGG_ENZYME:2.4.1.12	*bcsABI/C*	Rest of the above polypeptide?
914578	918165	3588	+	ID=fig|6666666.459278.peg.987;Name=hypothetical protein (Similar to bcsC)	*bcsCI/N*	Truncated form N-terminus
918140	918487	348	+	ID=fig|6666666.459278.peg.988;Name=Cellulose synthase operon protein C	*bcsCI/C*	Rest of the above polypeptide
918487	918957	471	+	ID=fig|6666666.459278.peg.989;Name=Cellulose synthase operon protein D	*bcsDI*	
919014	919163	150	+	ID=fig|6666666.459278.peg.990;Name=hypothetical protein	*orf990*	
919350	919565	216	+	ID=fig|6666666.459278.peg.991;Name=hypothetical protein	*orf991*	
919611	919739	129	+	ID=fig|6666666.459278.peg.992;Name=hypothetical protein	*orf992*	
919788	922007	2220	+	ID=fig|6666666.459278.peg.993;Name=beta-glucosidase (EC 3.2.1.21);Ontology_term=KEGG_ENZYME:3.2.1.21	*bglX*	
922010	922723	714	+	ID=fig|6666666.459278.peg.994;Name=Tiorf138 protein	*orf994*	
922746	922874	129	+	ID=fig|6666666.459278.peg.995;Name=hypothetical protein	*orf995*	
922956	923105	150	-	ID=fig|6666666.459278.peg.996;Name=hypothetical protein	*orf996*	
923165	923683	519	+	ID=fig|6666666.459278.peg.997;Name=FIG018171: hypothetical protein of Cupin superfamily	*orf997*	
923683	924333	651	+	ID=fig|6666666.459278.peg.998;Name=hydrolase%2C NUDIX family	*orf998*	
924459	926327	1869	+	ID=fig|6666666.459278.peg.999;Name=Chaperone protein HtpG	*htpG*	
1923097	1927566	4470	+	ID=fig|6666666.459278.peg.2003;Name=Cellulose synthase catalytic subunit [UDP-forming] (EC 2.4.1.12);Ontology_term=KEGG_ENZYME:2.4.1.12	*bcsABII_1*	
1927583	1931389	3807	+	ID=fig|6666666.459278.peg.2004;Name=hypothetical protein (Simiar to bcsC)	*bcsCII_1*	
3148370	3153238	4869	+	ID=fig|6666666.459278.peg.3276;Name=Cellulose synthase catalytic subunit [UDP-forming] (EC 2.4.1.12);Ontology_term=KEGG_ENZYME:2.4.1.12	*bcsABII*	
3153342	3154016	675	+	ID=fig|6666666.459278.peg.3277;Name=hypothetical protein (Similar to bcsX)	*bcsX*	
3154102	3155253	1152	+	ID=fig|6666666.459278.peg.3278;Name=O-antigen acetylase (Similar to bcsY)	*bcsY*	
3155288	3156388	1101	+	ID=fig|6666666.459278.peg.3279;Name=hypothetical protein (Similar to bcsC)	*bcsCII_2/N*	Truncated form N-terminus
3156435	3160727	4293	+	ID=fig|6666666.459278.peg.3280;Name=hypothetical protein (Similar to bcsC)	*bcsCII_3*	

E25 strain

Start	Stop	Length	Strand	Annotation	Gene name	Comments
472295	473245	951	+	ID=fig|6666666.458228.peg.503;Name=beta-1%2C4-glucanase (cellulase) (EC 3.2.1.4);Ontology_term=KEGG_ENZYME:3.2.1.4	*bcsZ*	
473326	474213	888	+	ID=fig|6666666.458228.peg.504;Name=hypothetical protein (similar to cellulose-complementing protein A)	*bcsH*	
474234	474353	120	+	ID=fig|6666666.458228.peg.505;Name=hypothetical protein	*orf505*	
474401	476638	2238	+	ID=fig|6666666.458228.peg.506;Name=Cellulose synthase catalytic subunit [UDP-forming] (EC 2.4.1.12);Ontology_term=KEGG_ENZYME:2.4.1.12	*bcsAI*	
476625	479060	2436	+	ID=fig|6666666.458228.peg.507;Name=hypothetical protein (Similar to bcsB)	*bcsBI*	
479063	483043	3981	+	ID=fig|6666666.458228.peg.508;Name=hypothetical protein (Similar to bcsC)	*bcsCI*	
483043	483513	471	+	ID=fig|6666666.458228.peg.509;Name=Cellulose synthase operon protein D	*bcsDI*	
483736	485940	2205	+	ID=fig|6666666.458228.peg.510;Name=beta-glucosidase (EC 3.2.1.21);Ontology_term=KEGG_ENZYME:3.2.1.21	*bglX*	
485949	486617	669	+	ID=fig|6666666.458228.peg.511;Name=Tiorf138 protein	*orf511*	
486691	487149	459	+	ID=fig|6666666.458228.peg.512;Name=FIG018171: hypothetical protein of Cupin superfamily	*orf512*	
487146	487775	630	+	ID=fig|6666666.458228.peg.513;Name=hypothetical protein	*orf513*	
488064	489950	1887	+	ID=fig|6666666.458228.peg.514;Name=Chaperone protein HtpG	*orf514*	
875669	876694	1026	-	ID=fig|6666666.458228.peg.905;Name=Endoglucanase	*bglC*	
2168861	2173441	4581	-	ID=fig|6666666.458228.peg.2304;Name=Cellulose synthase catalytic subunit [UDP-forming] (EC 2.4.1.12);Ontology_term=KEGG_ENZYME:2.4.1.12	*bcsABII_1*	
2521747	2524698	2952	-	ID=fig|6666666.458228.peg.2648;Name=hypothetical protein (Similar to bcsC)	*bcsCII/N*	Truncated form N-terminus
2524762	2525496	735	-	ID=fig|6666666.458228.peg.2649;Name=Transposase (Similar to IS5-like element IS1031A family transposase)	*IS_1*	
2525630	2526424	795	-	ID=fig|6666666.458228.peg.2650;Name=hypothetical protein (Similar to bcsC)	*bcsCII/C1*	Rest of the above polypeptide 1
2526433	2526687	255	-	ID=fig|6666666.458228.peg.2651;Name=hypothetical protein (Similar to bcsC)	*bcsCII/C2*	Rest of the above polypeptide 2
2526677	2527837	1161	-	ID=fig|6666666.458228.peg.2652;Name=hypothetical protein (Similar to bcsY)	*bcsY*	
2527921	2528592	672	-	ID=fig|6666666.458228.peg.2653;Name=hypothetical protein(Similar to bcsX)	*bcsX*	
2528730	2533283	4554	-	ID=fig|6666666.458228.peg.2654;Name=Cellulose synthase catalytic subunit [UDP-forming] (EC 2.4.1.12);Ontology_term=KEGG_ENZYME:2.4.1.12	*bcsABII_2*	
